# Pcf11 orchestrates transcription termination pathways in yeast

**DOI:** 10.1101/gad.251470.114

**Published:** 2015-04-15

**Authors:** Pawel Grzechnik, Michal Ryszard Gdula, Nick J. Proudfoot

**Affiliations:** 1 Sir William Dunn School of Pathology, University of Oxford, Oxford OX1 3RE, United Kingdom;; 2 Department of Biochemistry, University of Oxford, Oxford OX1 3QU, United Kingdom

**Keywords:** transcription termination, NRD complex, CTD phosphorylation, cleavage and polyadenylation complex, noncoding RNA

## Abstract

In *Saccharomyces cerevisiae*, short noncoding RNA (ncRNA) generated by RNA polymerase II (Pol II) are terminated by the NRD complex consisting of Nrd1, Nab3, and Sen1. We now show that Pcf11, a component of the cleavage and polyadenylation complex (CPAC), is also generally required for NRD-dependent transcription termination through the action of its C-terminal domain (CTD)-interacting domain (CID). Pcf11 localizes downstream from Nrd1 on NRD terminators, and its recruitment depends on Nrd1. Furthermore, mutation of the Pcf11 CID results in Nrd1 retention on chromatin, delayed degradation of ncRNA, and restricted Pol II CTD Ser2 phosphorylation and Sen1–Pol II interaction. Finally, the *pcf11-13* and *sen1-1* mutant phenotypes are very similar, as both accumulate RNA:DNA hybrids and display Pol II pausing downstream from NRD terminators. We predict a mechanism by which the exchange of Nrd1 and Pcf11 on chromatin facilitates Pol II pausing and CTD Ser2-P phosphorylation. This in turn promotes Sen1 activity that is required for NRD-dependent transcription termination in vivo.

## Abstract (precis)

In *Saccharomyces cerevisiae*, short noncoding RNA (ncRNA) generated by RNA polymerase II (Pol II) are terminated by the NRD complex consisting of Nrd1, Nab3, and Sen1. Grzechnik et al. show that Pcf11, a component of the cleavage and polyadenylation complex (CPAC), is generally required for NRD-dependent transcription termination through the action of its CTD-interacting domain (CID). Mutation of the Pcf11 CID results in Nrd1 retention on chromatin, delayed degradation of ncRNA, and restricted Pol II CTD Ser2 phosphorylation and Sen1–Pol II interaction.

RNA polymerase II (Pol II) in budding yeast uses two major pathways to terminate transcription. Poly(A) site (PAS)-dependent termination mediated by the cleavage and polyadenylation complex (CPAC) is associated with protein-coding genes (Mischo and Proudfoot 2012). Alternatively, NRD-dependent termination is specific for short noncoding RNA (ncRNA), including most small nucleolar RNA (snoRNA), cryptic unstable transcripts (CUTs), many stable unannotated transcripts (SUTs), and Xrn1-sensitive unstable transcripts (XUTs). NRD may also regulate hundreds of protein-coding genes through the promotion of premature transcription termination (Steinmetz et al. 2001, 2006b; Arigo et al. 2006a,b; Thiebaut et al. 2006; Kuehner and Brow 2008; Schulz et al. 2013; Webb et al. 2014). NRD comprises a heterodimer of Nrd1 and Nab3 associated with the RNA:DNA helicase Sen1 (Conrad et al. 2000; Vasiljeva and Buratowski 2006). Nrd1 and Nab3 RNA recognition motifs (RRMs) target specific RNA sequences (NRD-binding sites [NBSs]) (Carroll et al. 2004, 2007; Creamer et al. 2011). In vitro data suggest that Sen1 loaded onto nascent RNA may translocate along the transcript and displace Pol II (Porrua and Libri 2013). Consistently, in vivo studies indicate that slow transcription rates, which create a “termination window,” may allow termination factors time to catch up with the transcribing Pol II elongation complex (Hazelbaker et al. 2012).

A general view of pathway choice for Pol II transcription termination is that termination factor assembly depends on Rpb1 C-terminal domain (CTD) phosphorylation (Gudipati et al. 2008; Vasiljeva et al. 2008). This acts to recruit complexes that respond to either PASs or NBSs containing nascent RNA. At an early stage in the transcription cycle, phosphorylation of CTD Ser5 favors recruitment of the NRD complex via its Nrd1 CTD-interacting domain (CID) (Gudipati et al. 2008; Vasiljeva et al. 2008; Kubicek et al. 2012). As transcription progresses, Ser5 is dephosphorylated and replaced by CTD Ser2 phosphorylation (Kim et al. 2010; Mayer et al. 2010; Tietjen et al. 2010). This promotes the binding of the CID-containing CPAC factor Pcf11 (Lunde et al. 2010). However, recruitment profiles of both Nrd1 and Pcf11 do not precisely follow CTD phosphorylation patterns (Kim et al. 2010). Thus, Nrd1 also binds a CTD that has Ser5-P combined with Ser2-P and Ser7-P marks (Vasiljeva et al. 2008; Kubicek et al. 2012). Similarly, Pcf11–CTD interaction is not solely restricted to Ser2 phosphorylated CTD, as it also binds Ser5–Ser2 phosphorylated and unphosphorylated Pol II (Barilla et al. 2001; Lunde et al. 2010).

In human cells, transcription termination of short ncRNA called PROMPTs, related to *Saccharomyces cerevisiae* CUTs/SUTs, is reported to be Ser2-P- and CPAC-dependent (Almada et al. 2013; Ntini et al. 2013). Similarly in *S. cerevisiae*, CPAC-mediated transcription termination of short NRD-dependent RNA has been widely described (Morlando et al. 2002; Ganem et al. 2003; Kim et al. 2006, 2010; Grzechnik and Kufel 2008; Haddad et al. 2011; Al Husini et al. 2013). The essential role of CPAC in transcription termination of ncRNA is reinforced by the fact that CTD Ser2-P and Ser5-P marks often overlap over NRD-dependent terminators close to the transcription start site (TSS) (Kim et al. 2010; Mayer et al. 2010; Tietjen et al. 2010; Bataille et al. 2012). Moreover, inactivation of Ser2 kinase Bur1 or Ctk1 results in transcription readthrough at NRD terminators (Tomson et al. 2012; Lenstra et al. 2013). Ser2-P marks are also required for Sen1–Pol II binding, which is necessary for NRD-dependent termination in vivo (Finkel et al. 2010; Chinchilla et al. 2012; Chen et al. 2014) but not in vitro (Porrua and Libri 2013).

Our analysis of NRD-dependent termination in effect clarifies its direct link with the CPAC component Pcf11. Genome-wide analysis shows that most ncRNA are affected by Pcf11 inactivation, indicating the joint termination requirement for Nrd1 and Pcf11. In particular, Pcf11 is recruited to NRD terminators in an Nrd1-dependent manner immediately downstream from the Nrd1-binding site, and this is then required for Nrd1 chromatin release and ncRNA degradation. Pcf11 so recruited promotes phosphorylation of CTD Ser2 residues, which is in turn required for Sen1 activation to complete the NRD termination process.

## Results

### Pcf11 is required for transcription termination of most noncoding genes

**Figure 1.**
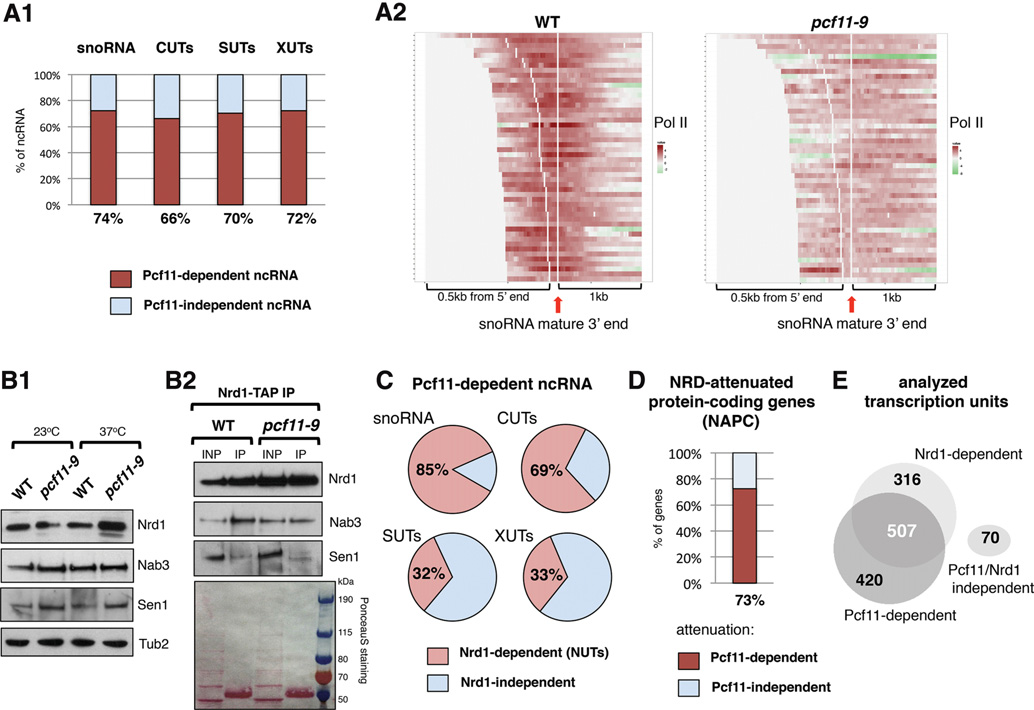
Pcf11 terminates the majority of ncRNA. (*A1*) Fraction of snoRNA, CUTs, SUTs, and XUTs that requires Pcf11 for transcription termination. (*A2*) Heat maps showing Pol II levels up to 0.5 kb 5′ and 1 kb 3′ of snoRNA mature 3′ ends in wild type (WT) and *pcf11-9*. Signals are aligned relative to snoRNA mature 3′ ends (vertical white line marked by red arrow). The white spacer for individual snoRNA marks the 5′ end. (*B1*) Western blot showing levels of NRD components in *pcf11-19*; anti-Nrd1, anti-Nab3, and anti-HA antibodies were used to detect Nrd1, Nab3, and Sen1-HA, respectively. (*B2*) Western blot analysis of fractions coprecipitated with Nrd1-TAP from wild type and *pcf11-9*. PAP antibody was used to detect Nrd1-TAP, while anti-Nab3 was used to detect Nab3, and anti-HA was used to detect Sen1-HA. (*C*) Overlap of Pcf11-dependent ncRNA with annotated NUTs. (*D*) Fraction of NAPC genes, which require Pcf11 for premature transcription termination. (*E*) Venn diagram presenting the overlap between Pcf11- and Nrd1-dependent transcription units.

To investigate the role of Pcf11 in transcriptional termination of ncRNA, we initially performed genome-wide analysis to establish generality. Pol II chromatin occupancy data have been previously described for *pcf11-9* mutant cells (Kim et al. 2010), which were shown to be defective in both PAS- and NRD-dependent termination (Amrani et al. 1997; Birse et al. 1998; Sadowski et al. 2003; Kim et al. 2006, 2010). However, we elected to further analyze this data set to specifically look for increased Pol II signal in downstream regions of NRD terminators relative to wild type (see the Materials and Methods; Supplemental Fig. S1 for details). All ncRNA that displayed termination defects were classified as “Pcf11-dependent.” Note that we discarded units with low Pol II signal. Seventy-four percent of snoRNA transcription units displayed a transcription termination defect in *pcf11-9*, as did 66% of CUTs, 70% of SUTs, and 72% of XUTs. These data are presented graphically (Fig. 1A1) and are also presented as heat maps for multiple transcription units (Fig. 1A2; Supplemental Fig. S2). We also established that the NRD complex is intact in *pcf11-9* cells based on protein levels of Nrd1, Nab3, and Sen1 as compared with wild-type cells (Fig. 1B1) and established their maintained interaction based on coimmunoprecipitation analysis (Fig. 1B2). This excludes indirect effects of Pcf11 inactivation on NRD complex integrity and argues that Pcf11 plays a more direct role in NRD-mediated termination. To further this view, we investigated the overlap of known Nrd1-dependent transcripts (Nrd1 unterminated transcripts [NUTs]) (Schulz et al. 2013) and Pcf11 requirement. Notably, we found that 85% and 69% Pcf11-dependent snoRNA and CUTs, respectively, were also affected by Nrd1 nuclear depletion (Fig. 1C). We surmise that for the majority of snoRNA and CUTs, neither NRD nor CPAC alone is able to mediate transcription termination, which argues against a redundant role for these separate termination complexes. Instead Pcf11 appears to directly cooperate with NRD termination. In contrast, SUTs and XUTs, previously reported to be more NRD-independent than snoRNA and CUTs (Marquardt et al. 2011; Schulz et al. 2013), rely significantly on CPAC for transcription termination, as only 32% and 33%, respectively (*P* < 10^−4^), were also classified as NUTs (Fig. 1C). Finally, we tested 30 out of 44 known NRD-attenuated protein-coding (NAPC) genes (Supplemental Table S5; Arigo et al. 2006a; Thiebaut et al. 2008; Creamer et al. 2011; Schulz et al. 2013) and found that for 22 such genes (73%), premature transcription termination was affected in *pcf11-9* (Fig. 1D; Supplemental Fig. S2D). Overall, for all 1313 analyzed transcription units, the majority of NRD-dependent genes was affected by both Nrd1 and Pcf11 inactivation. Only 316 transcription units were responsive to the NRD complex but did not display significant termination defects in *pcf11-9* and thus were not classified as “Pcf11-dependent” (Fig. 1E).

**Figure 2.**
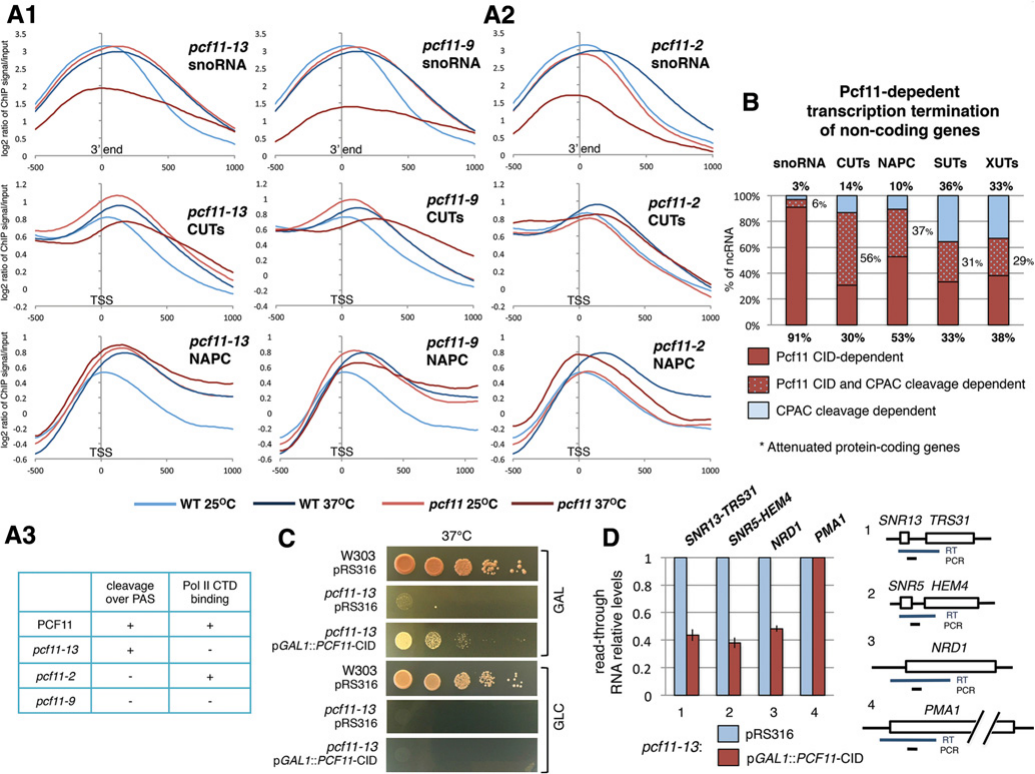
Pcf11 CID regulates NRD-dependent transcription termination. (*A1*,*A2*) Metagene analysis of Pol II occupancy in various *PCF11* mutants. Pol II signal was aligned to the snoRNA 3′ mature end or the TSS of CUTs and NAPC genes. Pol II was precipitated with the anti-CTD antibody CMA601. Plots present ChIP-seq signals smoothed with a 220-nucleotide (nt) moving average. (*A3*) Phenotypes of *PCF11* mutants (Amrani et al. 1997; Sadowski et al. 2003). (*B*) Transcription termination pathway distribution for Pcf11-dependent genes. (*C*) Suppression of temperature-sensitive *pcf11-13* growth by the episomal Pcf11 CID at 37°C. Expression of the CID was controlled by the *GAL1* promoter. Dilutions (1:10) were plated on YPGAL (inducing condition) or YPD (repressing condition) and grown for 4 d. (*D*) Accumulation of aberrant readthrough transcripts in *pcf11-13* detected following episomal expression of the Pcf11 CID by RT-qPCR analysis. Levels of readthrough transcripts were normalized to *PMA1* mRNA. Diagrams show reverse-transcribed regions and positions of qPCR amplicons. pRS316 was the control empty vector.

A limitation of the above analysis on *pcf11-9* is that the protein's CID and domains required for CPAC PAS cleavage are both inactivated. Furthermore, previous studies have indicated that these different domains may have differential importance in PAS-dependent as compared with NRD transcription termination (Birse et al. 1998; Sadowski et al. 2003; Kim et al. 2006). We therefore elected to generate new Pol II chromatin immunoprecipitation (ChIP) combined with deep sequencing (ChIP-seq) data using *pcf11* mutant strains that are selectively inactivated in specific domains: *pcf11-13* (three point mutations within the CID domain), lacking CTD-binding capacity, and *pcf11-2* (point mutations in the Q-rich segment, Rna14/Rna15-interacting domain, and zinc finger motifs), impaired in PAS-associated cleavage (Amrani et al. 1997; Sadowski et al. 2003). To allow consistent comparisons between data sets, we also performed a new Pol II ChIP-seq analysis on *pcf11-9*. Metagene profiles (Fig. 2A) were obtained for snoRNA, CUTs, and NAPC genes in each mutant strain compared with isogenic wild type and were grown at either permissive (25°C) or restrictive (37°C) temperatures. Remarkably, wild-type strains revealed a change in Pol II occupancy between 25°C and 37°C, indicating a termination defect (Fig. 2A, light- and dark-blue curves). At the elevated temperature, Pol II profiles shifted in a 3′ direction relative to the mature 3′ end (snoRNA) or TSS (CUTs and NAPC genes). This effect may be caused by faster transcription at 37°C (Miguel et al. 2013) and is consistent with the postulated “termination window” model, which assumes that NRD slow transcription rates promote earlier termination events (Hazelbaker et al. 2012). This may also indicate that distal regions of NRD terminators are more efficient than proximal elements. Consistently, this effect was not observed for SUTs and XUTs, which, as shown above, mainly use the CPAC termination pathway (Fig. 1C; Supplemental Fig. S3). Metagene analysis of the two mutant stains lacking CID function (*pcf11-13* and *pcf11-9*) showed a clear termination defect for snoRNA, CUTs, and NAPC genes even at 25°C (Fig. 2A1, light-red curves), similar to wild type at 37°C. At the higher nonpermissive 37°C temperature (Fig. 2A1, dark-red curves), even stronger termination defects were observed. However, the level of Pol II was overall lower for snoRNAs, indicating increased temperature sensitivity. Notably for *pcf11-2*, no termination defects were evident for these three gene classes (Fig. 2A2). These data confirm the dominant role of the Pcf11 CID in NRD-dependent termination.

Next, we assessed what proportion of Pcf11-dependent ncRNAs are differently affected by either Pcf11 CID (*pcf11-13*) or CPAC-mediated cleavage domain (*pcf11-2*) mutation (Fig. 2B). Transcription termination of independently transcribed snoRNA was predominantly (91%) Pcf11 CID-dependent. For CUTs, Pcf11 CID dependency was higher than cleavage dependency (30% and 14%, respectively; *P* = 0.021). However, most (56%) displayed a termination defect in either mutant. We also tested 19 NAPC genes, and 10 lost NRD attenuation in *pcf11-13*, but only two lost NRD attenuation in *pcf11-2*, with seven genes affected in both mutants. As expected, the largely CPAC-dependent SUTs and XUTs showed no bias toward the Pcf11 CID-mediated mechanism (Fig. 2B).

Previously, the Pcf11 CID has been suggested to be required but not sufficient for Pol II transcriptional termination (Sadowski et al. 2003). Consistent with this, we observed that episomal expression of the Pcf11 CID from the *GAL1* promoter only partially rescued *pcf11-13* temperature-sensitive growth (Fig. 2C). This indicates that Pcf11 CID function is not fully separate from the rest of the protein even though it may have an independent role in NRD-dependent termination. To confirm this, we analyzed readthrough transcription of selected NRD-dependent and Pcf11 CID-dependent but CPAC cleavage-independent genes (Schulz et al. 2013; data not shown) in a *pcf11-13* derivative strain that overexpresses Pcf11 CID. The genes tested were boxC/D snoRNA *SNR13*, boxH/ACA snoRNA *SNR5*, and the NAPC gene *NRD1*. RT-qPCR analysis showed that overexpression of the Pcf11 CID from the plasmid reduced accumulation of readthrough RNA, indicating a rescue of the *pcf11-13* termination defect (Fig. 2D). Overall, our data suggest that the function of Pcf11 in the NRD-dependent termination pathway is largely restricted to its CID–CTD interaction.

### Associated recruitment of Nrd1 and Pcf11 on NRD-dependent genes

**Figure 3.**
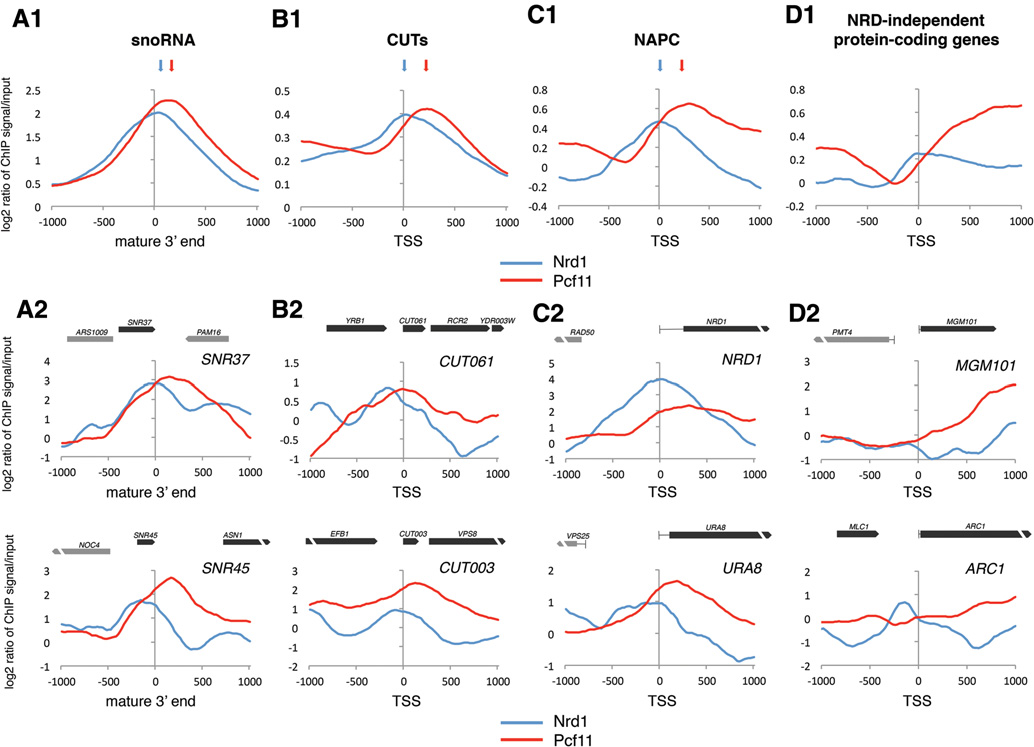
Differential localization of Nrd1 and Pcf11 over NRD terminators. (*A1*,*B1*,*C1*,*D1*) Metagene analysis of Nrd1 and Pcf11 recruitment relative to the mature 3′ ends of independently transcribed snoRNA (*A1*), CUT TSSs (*B1*), NAPC gene TSSs (*C1*), or Nrd1-independent protein-coding gene TSSs (*D1*). Blue and red arrows indicate the maximum signal for Nrd1 or Pcf11, respectively. (*A2*,*B2*,*C2*,*D2*) Pcf11 and Nrd1 recruitment over single genes. The *X*-axis marks the snoRNA mature 3′ end or either CUT or protein-coding gene TSSs. Plots are ChIP–chip signals smoothed with a 220-nt moving average.

It has been proposed that both Nrd1 and Pcf11 are recruited to NRD termination signals and compete for CTD binding in vivo (Singh et al. 2009; Kim et al. 2010). Therefore, to understand the role of Pcf11 CID–CTD interaction in NRD termination, we investigated Pcf11 interplay with Nrd1 on NRD-dependent genes by analyzing available genomic data sets of Nrd1 and Pcf11 chromatin association (Kim et al. 2010). First, we compared Nrd1 and Pcf11 recruitment over independently transcribed snoRNA transcription units (Fig. 3A1; Supplemental Table S5). Metagene analysis shows that Nrd1 peaks in position from −20 to +40 relative to the 3′ end and then gradually decreases. In contrast, Pcf11 binding only partially overlaps with Nrd1, being clearly 3′-shifted, reaching a maximum of 120–160 nucleotides (nt) downstream from the mature snoRNA 3′ end (*P* = 2.5 × 10^−4^). Analysis of Nrd1 and Pcf11 recruitment over CUTs (Xu et al. 2009) revealed similar differential distribution (Fig. 3B1; Supplemental Table S5). Nrd1 was recruited earlier than Pcf11, with a maximum level over the TSS. In contrast, Pcf11 peaked 200–220 nt downstream (*P* = 5 × 10^−3^). The same pattern was repeated for 44 analyzed NAPC genes (Fig. 3C1; Supplemental Table S5). Here, Nrd1 was mainly recruited close to the TSS (±100 nt), while Pcf11 distributed 160–360 nt downstream from TSS (*P* = 10^−4^). Analysis of single-gene examples confirmed the differences in Nrd1 and Pcf11 recruitment over ncRNA and protein-coding genes (Fig. 3A2,B2,C2). Remarkably, Nrd1 binding precisely overlapped with the regions where Pol II terminated at 25°C, while Pcf11 was recruited to the regions where Pol II was released at 37°C (Fig. 2A). We also compared Nrd1 and Pcf11 localization on NAPC genes with 40 randomly selected, actively transcribed, NRD-independent protein-coding genes. These later genes generally showed lower Nrd1 levels over the promoter and 5′ end, while Pcf11 was recruited at a later stage of transcription than for NAPC genes (Fig. 3D1,D2; Supplemental Fig. S4).

**Figure 4.**
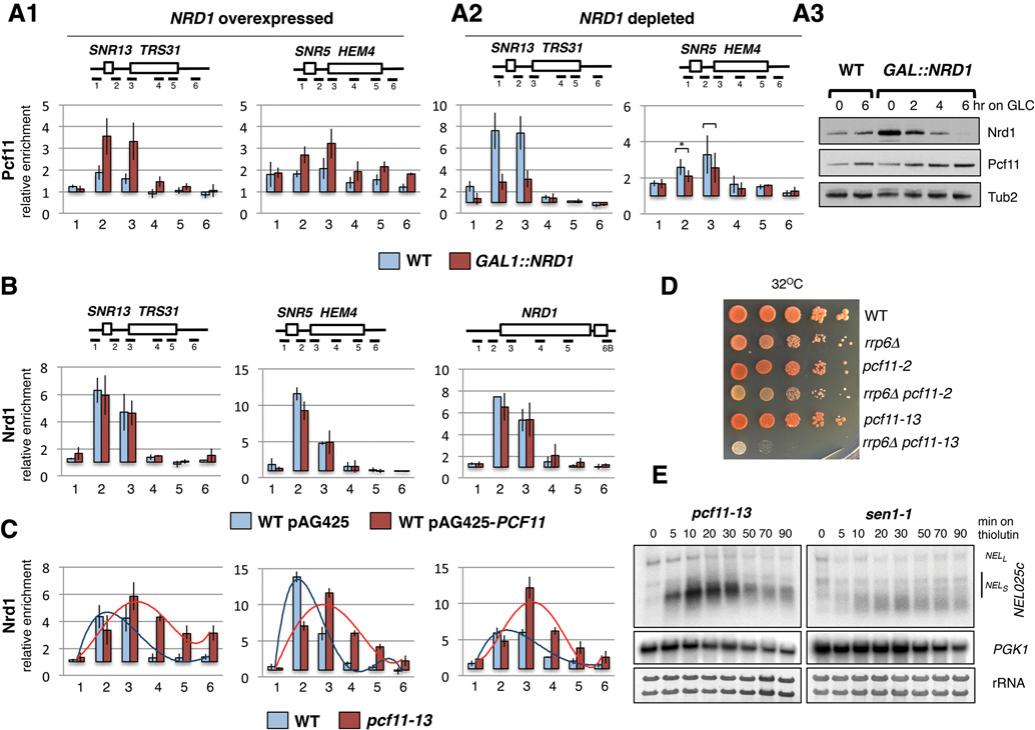
Interdependence between Nrd1 and Pcf11 recruitment over NRD-dependent genes. ChIP analysis on the indicated genes of Pcf11 recruitment in Nrd1-overexpressed (*A1*) and Nrd1-depleted (*A2*) cells. Expression of *GAL1:NRD1* was shut down for 6 h at 30°C prior to analysis. (*A3*) Western blot showing levels of Nrd1 and Pcf11 following either *NRD1* overexpression or depletion. Anti-Nrd1, anti-HA, and anti-Tub2 antibodies were used to detect Nrd1, Pcf11-HA, and Tub2, respectively. (*B*) ChIP analysis on the indicated genes of Nrd1-TAP recruitment in cells overexpressing Pcf11; cells transformed with the respective plasmids were grown in SD −ura at 30°C. (*C*) ChIP analysis on the indicated genes (as for *B*) of Nrd1 recruitment in *pcf11-13*. Trend lines emphasize Nrd1 profiles. (*D*) Growth tests of the *rrp6Δ pcf11-13* strain at an elevated temperature. Dilutions (1:10) were grown for 4 d at 32°C on SD medium. (*E*) Northern blots showing transcriptional shutoff analysis of *NEL025c* ncRNA in *pcf11-13* and *sen1-1*. Cells were incubated at the nonpermissive temperature for 30 min prior to thiolutin-specific transcription inhibition. *NEL025c* long (*NEL*_*L*_) and short (*NEL*_*S*_) forms are indicated. *PGK1* mRNA and rRNA were used as degradation and loading controls. Diagrams *above* the graphs depict loci and positions of amplicons. The box at the 3′ end of *NRD1* represents the TAP tag. The average of three experiments showing fold enrichment over the background control from a nontranscribed region on Chromosome V (>1) is shown. Error bars represent standard deviation of repeats.

Our genomic analyses show that Nrd1 is recruited in proximal regions of NRD-dependent terminators, while Pcf11 is localized immediately downstream from Nrd1-binding regions. It therefore seems unlikely that Nrd1 and Pcf11 compete for recruitment over NRD terminators. To confirm this, we used ChIP to analyze Nrd1 and Pcf11 recruitment under varying expression levels of each factor. First, we analyzed Pcf11 recruitment in a *GAL1::NRD1* strain grown in galactose (Fig. 4A1). The *GAL1* untranslated region (UTR) overrides *NRD1* premature transcription termination and so results in Nrd1 accumulation (Fig. 4A3, time point 0). Remarkably, under these Nrd1 overexpression conditions, rather than competitive binding, we observed increased recruitment of Pcf11 over *SNR13* and *SNR5*, which peaked over amplicons 2 and 3, known to be NRD terminators for *SNR13* and *SNR5* (Steinmetz and Brow 2003; Steinmetz et al. 2006a; Singh et al. 2009). Detailed genomic features of the analyzed units are depicted in Supplemental Figure S5. Next, we investigated Pcf11 recruitment in the absence of Nrd1 (Fig. 4A2) by repressing *GAL1::NRD1* expression by growth in glucose-containing medium (Fig. 4A3). ChIP analysis revealed that following Nrd1 depletion, Pcf11 recruitment was inhibited over NRD terminators of *SNR13* (amplicons 2 and 3). A similar although weaker effect was observed over *SNR5*. Note that under these glucose growth conditions, there is higher Pcf11 occupancy over the NRD terminators tested in wild type (especially for *SNR13*).

Next, we examined whether Pcf11 influences Nrd1 recruitment. We overexpressed Pcf11 from a multicopy plasmid in wild-type cells (Supplemental Fig. S6A) and analyzed Nrd1 recruitment using a *NRD1::TAP* strain. Pcf11 has been previously reported to reduce levels of excessively recruited Nrd1 in the *ess1*^*H164R*^ mutant (Singh et al. 2009). However, no significant alternation of Nrd1 profiles over *SNR13*, *SNR5*, or *NRD1* was detected in wild-type overexpressing Pcf11 (Fig. 4B; Supplemental Fig. 6A). This argues that the initial Nrd1 recruitment is independent of Pcf11 levels. We also assessed whether loss of the function of the Pcf11 CID affects Nrd1 recruitment. ChIP analysis revealed that the Nrd1 recruitment profile was significantly altered in *pcf11-13* grown at the nonpermissive temperature (37°C) (Fig. 4C; Supplemental Fig. S6B). In this mutant strain, Nrd1 levels over *SNR13* and *SNR5* strongly accumulated at amplicon 3, downstream from the proximal NBS region, and then gradually reduced toward the downstream PAS. A similar pattern was repeated for *NRD1*. These results indicate that loss of Pcf11–CTD interaction alters Nrd1 recruitment patterns by allowing its accumulation over more distal parts of NRD terminators. This delayed Nrd1 recruitment profile in *pcf11-13* was not due to a general termination defect over NRD-dependent genes. Thus, the Nrd1 recruitment profile over the same gene loci in the *sen1-1* mutant (point mutation in motif IV of the helicase domain 2) (Ursic et al. 1997) showed overall elevated Nrd1 levels but no relative downstream shift in Nrd1 binding (Supplemental Fig. S6C1,2). Our analysis of Nrd1 and Pcf11 interplay over NRD-dependent genes indicates that Nrd1 binding stimulates Pcf11 recruitment in the early stages of transcription. In turn, Pcf11–CTD interaction prevents the spread of Nrd1 binding into terminator-distal regions.

The Nrd1 CID is known to affect RNA degradation, as it interacts directly with Trf4 and so couples the nuclear exosome with nascent RNA (Vasiljeva and Buratowski 2006; Kubicek et al. 2012; Heo et al. 2013; Tudek et al. 2014). We therefore investigated the possibility that the observed aberrant Nrd1 recruitment in *pcf11-13* may affect RNA turnover. Growth of the *pcf11-13*/*rrp6Δ* double mutant was severely affected at subpermissive (32°C), permissive, and nonpermissive temperatures, while *pcf11-2* had no synthetic effect with *RRP6* deletion (Fig. 4D; Supplemental Fig. S7A). This phenotype was not due to a general NRD termination defect in the *rrp6Δ* background, as *sen1-1* combined with *RRP6* deletion did not affect growth (Supplemental Fig. S7B). Similarly, loss of Rrp6 did not reduce the growth rate of *clp1-12* (Supplemental Fig. S7C), another CFIA mutant, defective in mRNA cleavage (Haddad et al. 2011).

To confirm a genetic interaction between Pcf11 and Rrp6, we assessed *CUT542* (*NEL025c*) decay in *pcf11-13* (Fig. 4E). Since Rrp6 rapidly degrades *NEL025c* in wild type, we compared its accumulation in *pcf11-13* with a control strain, *sen1-1*. When NRD-dependent transcription termination is affected, *NEL025c* short (*NEL*_*S*_) and long (*NEL*_*L*_) forms are detectable (Thiebaut et al. 2006). To investigate their decay, we inhibited transcription with thiolutin and measured *NEL025c* levels over time. Significantly, in *pcf11-13*, but not in *sen1-1*, *NEL*_*S*_ accumulated in the first 10 min of inhibition and then subsequently decreased. The readthrough product *NEL*_*L*_ decreased gradually in time in both *pcf11-13* and *sen1-1*. NRD-independent *PGK1* was similarly degraded in both mutants (Fig. 4E; Supplemental Fig. S7D). The *rrp6Δ* enhanced *pcf11-13* temperature-sensitive phenotype and perturbed CUT degradation in *pcf11-13* suggest an additional link between Pcf11 and the NRD-dependent pathway by showing a Pcf11 CID role in the processing and degradation of NRD-dependent RNA.

### Pcf11 affects CTD Ser2 phosphorylation

**Figure 5.**
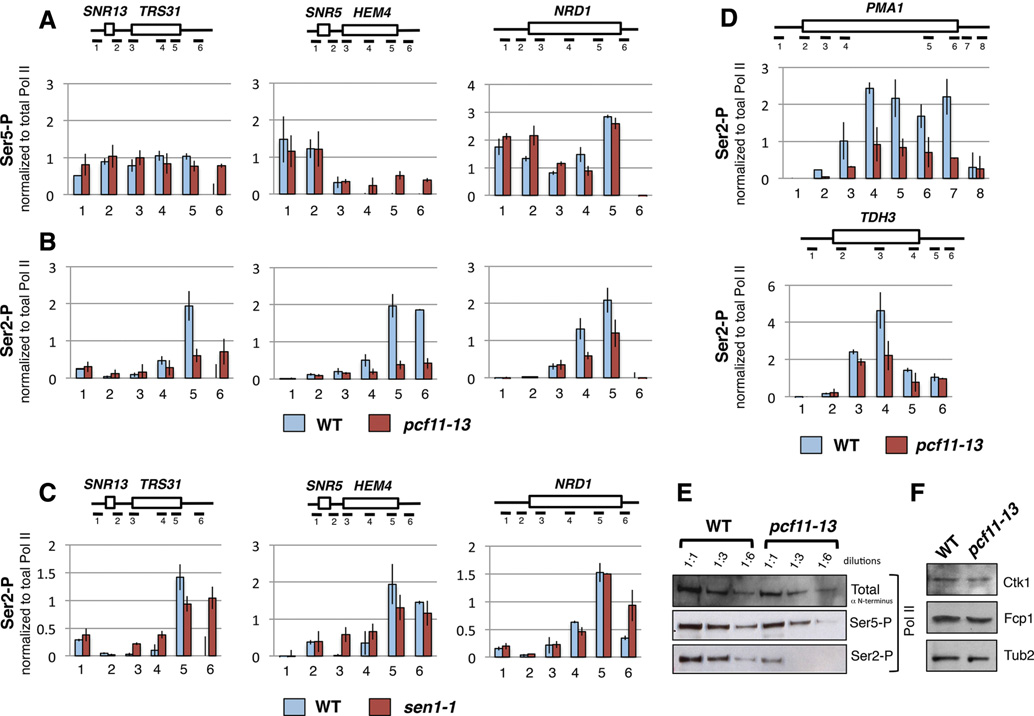
Lack of Pcf11–CTD interaction modulates CTD Ser2 phosphorylation. ChIP analyses of Pol II CTD Ser5-P (*A*) and Ser2-P (*B*) over selected NRD-dependent loci in *pcf11-1*3 cells. (*C*) Accumulation of Ser2-P in the *sen1-1* mutant over the same NRD-dependent genes. (*D*) Ser2-P over NRD-independent *PMA1* and *TDH3* in *pcf11-13*. Signals originating from phospho-CTD were normalized to total Pol II level detected over the indicated amplicons. Averages from three biological repeats are shown; error bars indicate standard deviation. Diagrams *above* the graphs depict loci and the locations of the amplicons. (*E*) Western blot analysis of total, Ser5, and Ser2 CTD phosphorylated Pol II in *pcf11-13*. Threefold dilutions of each protein extracts were blotted as indicated. PonceauS staining relative to Ser2-P detection is shown in Supplemental Figure S8D. (*F*) Western blot showing levels of CTD Ser2 kinase Ctk1 and CTD Ser2 phosphatase Fcp1 in *pcf11-13*. Tub2 was used as a loading control.

Enhanced Nrd1 recruitment over snoRNA genes was previously described for the *ess1*^*H164R*^mutant, which is also deficient in CTD Ser5-P dephosphorylation (Singh et al. 2009). We therefore investigated whether disturbance of Nrd1–Pcf11 exchange on chromatin, as we observed in *pcf11-13*, correlates with Pol II CTD phosphorylation. To evaluate differences in CTD phosphorylation between wild type and *pcf11-13*, we normalized the ChIP signal of phosphorylated Pol II to total Pol II levels. Our analyses of Pol II phosphorylated at Ser5 did not show any clear or consistent changes between wild type and *pcf11-13* over *SNR13-TRS31*, *SNR5-HEM4*, and *NRD*1 (Fig. 5A). In contrast, *pcf11-13* showed substantially reduced Ser2 phosphorylation over these transcription units, up to six times lower than in wild type (Fig. 5B). Note that some Ser2 phosphorylated Pol II was detected over NBSs (Supplemental Fig. S8A), as previously reported (Kim et al. 2010; Mayer et al. 2010; Tietjen et al. 2010; Bataille et al. 2012). To ensure that the observed phenotype was not a general feature of an NRD-dependent transcription termination defect, we investigated Pol II Ser2 phosphorylation in *sen1-1*. In contrast to *pcf11-13,* Ser2-P marks were less affected in this mutant (Fig. 5C). To determine whether the influence of Pcf11 on Ser2-P levels is specific for NRD-dependent genes, we also examined CTD phosphorylation over NRD-independent *PMA1* and *TDH3*. Although levels of Pol II transcribing both genes was comparable between wild type and the mutant (Supplemental Fig. S8B), Ser2-P marks were again strongly reduced in *pcf-11-13* (Fig. 5D; Supplemental Fig. S8C).

Global changes in Pol II Ser2 phosphorylation in *pcf11-13* were confirmed by Western blot (Fig. 5E). Thus, serial dilutions of protein extracts from wild type and *pcf11-13* (Supplemental Fig. S8D) were analyzed with antibody against the Rpb1 N terminus and Ser5-P-specific or Ser2-P-specific antibodies. Ser2 phosphorylated Pol II was clearly depleted from the *pcf11-13* extract. The signal was significantly decreased at the first dilution as compared with wild type and at the background levels in the following dilutions. Note that levels of enzymes regulating CTD Ser2 phosphorylation (Ctk1 and Fcp1) (Cho et al. 2001) were not affected in *pcf11-13* (Fig. 5F). Overall these results indicate a general mechanism of Ser2 phosphorylation regulation through Pcf11–CTD interaction. We therefore next considered the association of Pcf11 with Sen1, which is known to interact with Ser2 phosphorylated Rpb1 (Chinchilla et al. 2012).

### Pcf11 promotes Sen1 function

**Figure 6.**
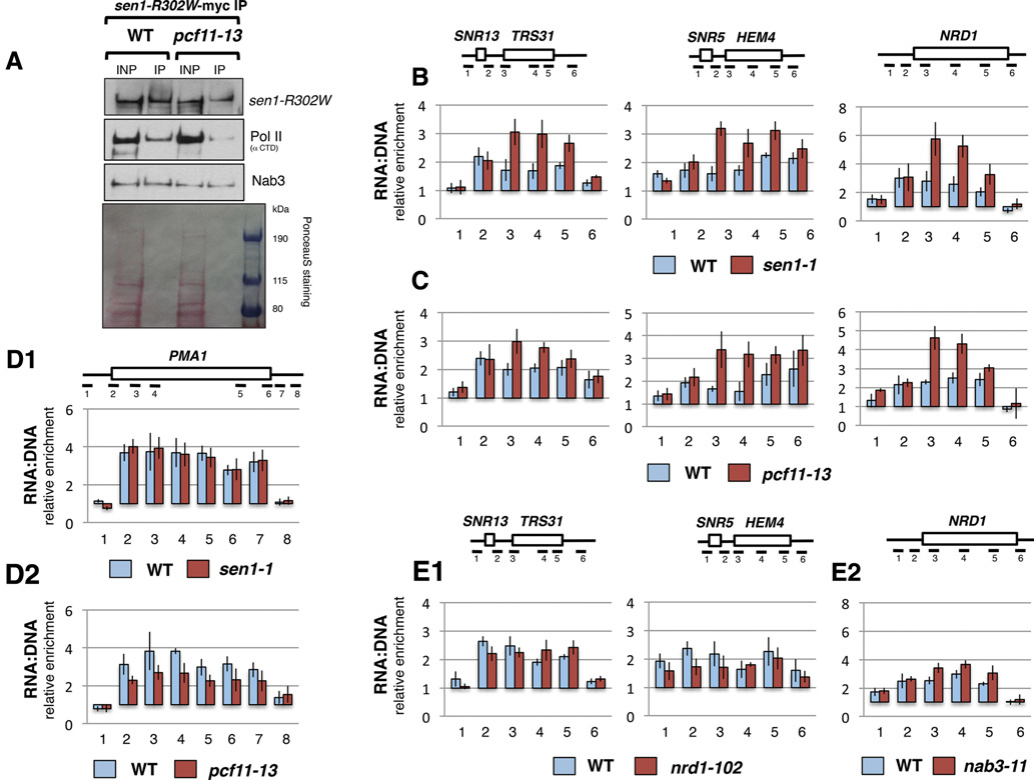
RNA:DNA hybrids accumulate downstream from the NBS in *sen1-1* and *pcf11-13*. (*A*) Analysis of Pol II–Sen1-R302W interactions in *pcf11-13* by Western blot. Sen1-R302W-myc was precipitated with anti-myc antibody. Coimmunoprecipitated Pol II was detected using anti-CTD antibody CMA601, and Nab3 was detected with anti-Nab3 antibody. For Sen1-R302W-myc analysis, immunoprecipitated fractions were diluted five times prior to Western blot. PonceauS staining shows membrane subjected to Sen1-R302W-myc detection. (*B*–*E*) DNA immunoprecipitation (DIP) analysis of RNA:DNA hybrid accumulation over the indicated NRD-dependent genes and *PMA1* in *sen1-1* (*B*,*D1*), *pcf11-13* (*C*,*D2*), *nrd1-102* (*E1*), and *nab3-11* (*E2*). For each mutant, isogenic wild type (WT) was used. Diagrams and ChIP standardization are as described for Figure 4. S9.6 antibody was used to precipitate RNA:DNA hybrids.

To investigate whether Pcf11 facilitates Sen1–Pol II contact and Sen1 function in NRD-dependent termination, we undertook two approaches. First, we tested Sen1–Pol II interaction under lowered CTD Ser2 phosphorylation in *pcf11-13*. We used the *sen1-R302W* mutant, which harbors a point mutation in the N terminus that weakens Sen1–Pol II interaction (Finkel et al. 2010; Chinchilla et al. 2012). Western blot analysis revealed reduced Pol II coimmunoprecipitated with Sen1-R302W in *pcf11-13*, while the level of Nab3 coimmunoprecipitation with Sen1-R302W was unaffected as compared with wild type (Fig. 6A). This experiment showed that Pol II CTD–Sen1-R302W interaction was additionally reduced in *pcf11-13*.

Second, we investigated whether *pcf11-13* affects Sen1 enzymatic activity in vivo. The helicase activity of Sen1 is thought to be involved in RNA:DNA hybrid unwinding (Mischo et al. 2011; Chan et al. 2014). Therefore, we tested whether RNA:DNA hybrids localize in a similar way in *sen1-1* and *pcf11-13* by using a modified ChIP protocol (DNA immunoprecipitation [DIP]) with the RNA:DNA-specific antibody S9.6. This antibody recognizes RNA:DNA duplexes of at least 15 nt in a sequence-independent fashion (Boguslawski et al. 1986). Inactivation of Sen1 activity or Pcf11–CTD interaction similarly affected the RNA:DNA hybrid profiles over NRD terminators, resulting in an accumulation downstream from the proximal NBS (from amplicon 3 and beyond) of *SNR13-TRS31*, *SNR5-HEM4*, and *NRD1* (Fig. 6B,C). To show that the observed RNA:DNA hybrid accumulation in *sen1-1* and *pcf11-13* correlated with the presence of NRD terminators, we tested the NRD-independent gene *PMA1*. Notably, we did not detect any change in hybrid accumulation between wild type and *sen1-1* (Fig. 6D1). For *pcf11-13*, we observed a general reduction of RNA:DNA hybrid levels (Fig. 6D2). We finally tested other NRD complex mutants—*nrd1-102* and *nab3-11* (point mutation and double point mutation in the RRM, respectively) (Conrad et al. 2000)—for RNA:DNA hybrid accumulation. No change in hybrid level was observed over *SNR13-TRS31*, while their accumulation was slightly lower over *SNR5-HEM4* in *nrd1-102* than in wild type (Fig. 6E1). The *nab3-11* mutant strain showed only a minor increase of RNA:DNA hybrids over *NRD1* in the middle of the gene (Fig. 6E2). Our data indicate that RNA:DNA hybrids accumulate downstream from proximal NBSs when either Sen1 activity or Pcf11–CTD interaction is inactivated.

### Pol II pauses downstream from NRD-dependent terminators in *sen1-1* and *pcf11-13* mutants

**Figure 7.**
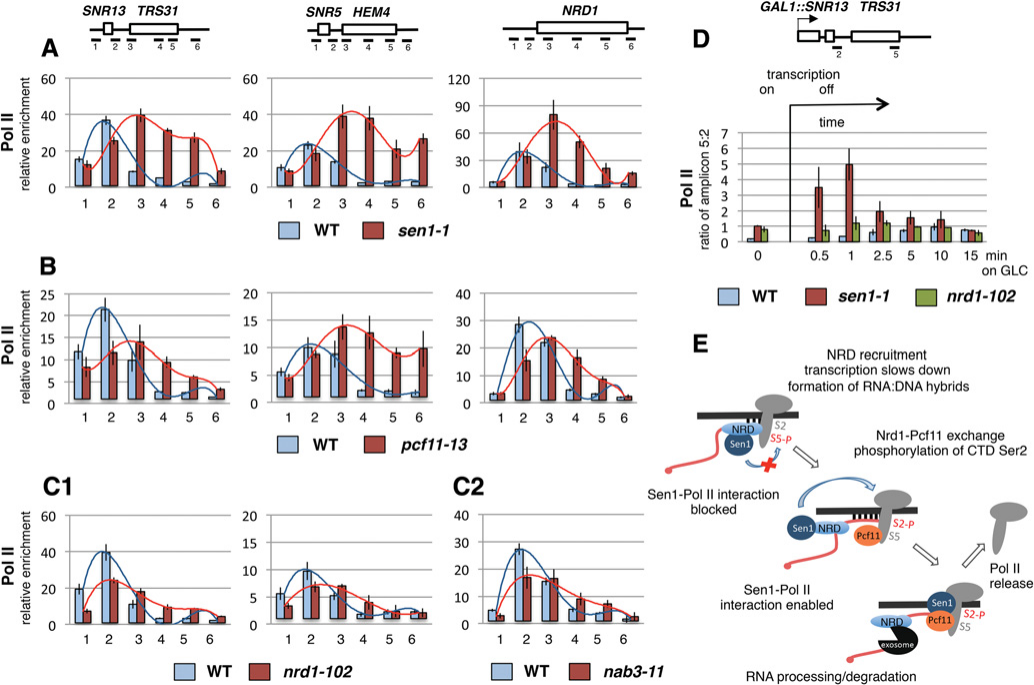
*pcf11-13* and *sen1-1* display similar accumulations of Pol II over NRD-dependent genes. (*A*–*C*) Pol II distribution over NRD-dependent genes in *sen1-1* (*A*), *pcf11-13* (*B*), *nrd1-102* (*C1*), and *nab3-1* (*C2*). (*D*) ChIP analysis showing Pol II kinetics of accumulation in wild type (WT), *sen1-1*, and *nrd1-102* on *GAL1::SNR13-TRS31* under induced and repressed conditions at the indicated times. Ratio of Pol II density over regions covered by amplicons 2 (NRD terminator) and 5 (TRS31 3′ end) are shown. Diagrams, ChIP procedure, and standardization are as described for Figure 4. The Pol II antibody was 8WG16. (*E*) A model of NRD-dependent transcription termination. (DNA) Thick black line; (RNA) red line.

RNA:DNA hybrids have been shown to relate to Pol II pausing in human cells (Skourti-Stathaki et al. 2011). Thus, to reinforce Sen1–Pcf11 association, we investigated whether RNA:DNA hybrid accumulation correlates with Pol II profiles (precipitated with 8WG16 antibody) in *sen1-1* and *pcf11-13* (Fig. 7A,B). Analyses of *sen1-1* and *pcf11-13* revealed that Pol II density decreased or remained the same over the proximal parts of snoRNA NRD terminators and the 5′ end of *NRD1* (amplicon 2) when compared with wild type. However, in the regions downstream, Pol II signals strongly accumulated in both mutants on all three genes. The values obtained for regions covered by amplicons 3 and 4 exceeded values from the upstream regions (amplicon 2), which indicates that Pol II that crossed proximal NBSs paused and piled up. Our data are consistent with previous results showing that Pol II accumulation shifts toward the 3′ ends in the *sen1-E1597K* mutant (Steinmetz et al. 2006b). To investigate whether observed phenotypes for *sen1-1* and *pcf11-13* are characteristic of other NRD complex mutants, we also performed Pol II ChIP analyses with *nrd1-102* and *nab3-11* (Fig. 7C1,C2). In these strains, although Pol II extended beyond the NRD terminators, its density did not exceed upstream values (cf. the ratio of amplicon 2 to 3 and 4). These results imply that Pol II accumulation downstream from the proximal NBS is a specific feature of Sen1 and Pcf11 inactivation rather than a general phenotype of termination-deficient NRD mutants.

To confirm that the observed Pol II accumulation downstream from the NBS in *sen1-1* and, by extension, *pcf11-13* is due to slow progression of polymerase across the downstream gene, we inserted the inducible *GAL1* promoter in place of the *SNR13* promoter and analyzed transcription kinetics over the *GAL1::SNR13-TRS31* unit (Fig. 7D; Supplemental Fig. S9). Following repression of the *GAL1* promoter with glucose, the speed of transcribing Pol II was determined by comparing the Pol II density between the the NRD terminator (amplicon 2) and the 3′ end of *TRS31* (amplicon 5). As a control for *sen1-1*, we used *nrd1-102* and wild type. In *sen1-1*, the amount of Pol II at the *TRS31* 3′ end increased five times as compared with ∼1.5-fold for *nrd1-102* after 1 min of repression. The Pol II level at the *TRS31* 3′ end was also higher after 2.5 min of repression. Finally, the ratio reached 1 as Pol II completed transcription of *TRS31* after 5–10 min in *sen1-1* and 2.5–5 min in *nrd1-102*. This experiment confirms that upon loss of Sen1 activity, Pol II pausing occurs downstream from the proximal regions of NRD terminators.

## Discussion

Our results clarify the role of Pcf11 in transcription termination of NRD-dependent genes and show its widespread involvement in the termination of ncRNA. Although Pcf11 also mediates the PAS/cleavage-dependent pathway for some ncRNA genes, its main function in the NRD-dependent pathway is to cooperate with Nrd1. We show that Pcf11 replaces Nrd1 on the CTD and so facilitates CTD phosphorylation at Ser2, which in turn promotes Sen1 function. ncRNA show termination defects either in Nrd1-depleted cells (Schulz et al. 2013) or upon Pcf11 inactivation (Kim et al. 2006, 2010). Strikingly, most snoRNA, CUTs, and NAPC genes require both Nrd1 and Pcf11 for transcription termination or attenuation (Fig. 1). Our data therefore establish that NRD and CPAC are not interchangeable at NRD-dependent genes, as their transcription termination is equally compromised by inactivation of either Nrd1 or Pcf11.

A cleavage-dependent PAS-like mechanism was previously suggested for ncRNA termination (Fatica et al. 2000; Morlando et al. 2002; Garas et al. 2008; Grzechnik and Kufel 2008; Kim et al. 2010; Al Husini et al. 2013). However, *SNR13* and *SNR33* were shown to require only the Pcf11 CID (Kim et al. 2006). Our results now demonstrate that mRNA-like CPAC-dependent transcription termination rarely acts on snoRNA. Instead, their termination is almost fully Pcf11–CID-dependent (Fig. 2A,B). Similarly, Pcf11–CID dependency is dominant for CUT and NAPC gene termination. However, for both groups, CPAC cleavage-dependent termination is used more frequently than for snoRNA. Such termination may occur on cryptic PASs, since the yeast PAS consensus sequence Py(A)_n_ is highly redundant (Mischo and Proudfoot 2012) and may be present more often in longer CUTs and attenuation regions than more compact snoRNA terminators. Cleavage-dependent termination is dominant over Pcf11–CID dependency for SUTs and XUTs (Fig. 2B), which correlates with known decreased NRD dependency in these groups (Schulz et al. 2013). Taking into account these relationships, we suggest that Pcf11 is a general component of the NRD termination pathway, where it acts in a CID-dependent manner, separate from its CPAC function.

Previous studies suggested that Pcf11 and Nrd1 chromatin association overlaps at NRD terminators (Kim et al. 2010). However, we now show that Pcf11 is recruited downstream from Nrd1-binding positions over distal NRD terminator regions (Fig. 3). Interestingly, even though Pcf11 and Nrd1 do not directly compete for CTD binding, their corecruitment is still interconnected. Thus, Pcf11 recruitment over NRD terminators increases upon Nrd1 overexpression and decreases upon Nrd1 depletion (Fig. 4A). Such a cooperative mechanism may facilitate Pcf11 recruitment at early stages of transcription. Similarly, at the 3′ ends of protein-coding genes, Pcf11 is recruited in a cooperative manner with another CID-containing protein, Rtt103 (Lunde et al. 2010). In contrast, Nrd1 recruitment is Pcf11-independent (Fig. 4B). Notably, Pcf11 acts to restrict Nrd1 over the distal regions of NRD terminators (Fig. 4C).

Unexpectedly, the Pcf11 CID mutation genetically interacts with the nuclear exosome cofactor exonuclease Rrp6. Deletion of *RRP6* was shown to alleviate the temperature-sensitive phenotype of mutation in another CFIA factor, *RNA14* (Torchet et al. 2002). In contrast, *rrp6Δ* strongly exacerbated the *pcf11-13* temperature-sensitive phenotype (Fig. 4D). Pcf11-dependent release of Nrd1 from the CTD may facilitate Nrd1 CID–Trf4 interaction and exosome recruitment (Tudek et al. 2014) and subsequent processing or degradation of ncRNA. Consistently, thiolutin-dependent transcriptional shutoff in *pcf11-13* was followed by an initially strong increase of ncRNA *NEL025c* and its subsequent delayed degradation. Thiolutin inhibits transcription initiation but has less effect on elongation (Tipper 1973). Therefore, the observed *NEL025c* accumulation may represent RNA associated with Nrd1 and transcribing Pol II, which is not degraded until Nrd1 is released from the CTD.

The importance of Pol II Ser2-P marks for NRD termination is underscored by the fact that mutations of the Ser2 kinases Ctk1 and Bur1 affect termination of NRD-dependent genes (Steinmetz et al. 2001; Tomson et al. 2012; Lenstra et al. 2013). Consistently, CTD Ser2 alanine substitutions also affect NRD-dependent termination (Lenstra et al. 2013). Our results reveal that Pcf11–CTD interaction enhances Ser2 phosphorylation levels (Fig. 5B,E). We predict that Pcf11 may enhance Ser2-P levels by its physical presence on the CTD, which may restrict phosphatase accessibility. Ser2-P marks are also needed for direct Sen1–Rpb1 interaction (Chinchilla et al. 2012), which is in turn crucial for transcription termination in vivo (Finkel et al. 2010; Chen et al. 2014). Indeed, we observed a decrease in Sen1–Pol II interaction in *pcf11-13* (Fig. 6A), suggesting that Pcf11 facilitates this contact. This is consistent with the crystal structure of the Pcf11 CID with CTD peptides that indicates Ser2-P accessibility for termination factors such as Sen1 (Meinhart and Cramer 2004).

Inactivation of Sen1 helicase activity in *sen1-1* resulted in accumulation of RNA:DNA hybrids (Mischo et al. 2011; Chan et al. 2014), especially over the distal parts of NRD terminators and in the regions downstream (Fig. 6B). As with *sen1-1*, we also detected accumulation of RNA:DNA hybrids in the same regions in *pcf11-13* (Fig. 6C). This indicates that Sen1 helicase activity is reduced upon Pcf11 CID mutation. We predict that a principal function of Pcf11 in the NRD pathway is to replace Nrd1 on the CTD and so allow Ser2 phosphorylation. This in turn enables Sen1–Rpb1 contact and Sen1 translocation that unwinds RNA:DNA hybrids behind Pol II. This model of Pcf11 function is reinforced by the fact that RNA:DNA hybrids accumulate over distal NRD terminator regions in *sen1-1* and *pcf11-13* and effectively colocalize with Pcf11 recruitment in wild type (Figs. 3, 6B,C).

RNA:DNA hybrids have been linked with transcription termination and Pol II pausing (Bentin et al. 2005; Bochkareva et al. 2011; Mischo et al. 2011; Skourti-Stathaki et al. 2011). Using ChIP analyses, we show that a similar association exists in *sen1-1* and *pcf11-13*, as both mutants display a phenotype in which Pol II accumulates over distal regions of NRD terminators (Fig. 7A,B). This suggests that, in these mutants, Pol II that has passed the proximal NBS pauses prior to release by Sen1 from the DNA template. This is consistent with previous studies showing that slow transcription rates or pausing are required for the Sen1-associated termination process (Hazelbaker et al. 2012). Possibly, the stepwise interaction of the Pol II CTD first with Nrd1 and then with Pcf11 may protect the CTD from elongation factor binding and favor transcription termination (“termination window”) (Hazelbaker et al. 2012) rather than continued elongation. Indeed, metagene analysis shows that Pol II termination of ncRNA in cells growing at 25°C overlapped with Nrd1 binding (Fig. 2A). However, at 37°C, snoRNA and CUT termination as well as protein-coding gene attenuation shifted to further downstream to the distal NRD terminator regions where Pcf11 reached its maximum. Elevated temperature increases the Pol II transcription rate (Miguel et al. 2013). Under such conditions, Sen1 may not be efficiently engaged over the proximal regions of NRD terminators and require higher Pcf11 concentration on chromatin to terminate transcription. This also suggests that the distal NRD regions are more efficient terminators than the proximal regions. Consistently, the same phenotype of termination and attenuation shift was repeated in *pcf11-13* at 25°C, at which the compromised Pcf11–CTD interaction was not sufficient to support early termination of slow transcribed Pol II (Fig. 2A).

Our data suggest a more complete model for the mechanism of NRD-dependent transcriptional termination (Fig. 7E). NBSs present on the nascent RNA and Ser5 phosphorylated CTD act to recruit the NRD complex. The simultaneous binding of NRD to Pol II and the nascent transcript bridge the RNA and DNA templates and so stimulate the formation of the RNA:DNA hybrid between the transcript and the complementary DNA strand. Nrd1 binding stimulates Pcf11 recruitment, which in turn releases Nrd1. Subsequent association of the CTD first with Nrd1 and then Pcf11 protects Pol II against the transition into the elongation phase and so restricts Pol II transcription. Pcf11 then facilitates stable CTD Ser2 phosphorylation, which promotes Sen1–Pol II interaction. Sen1 then translocates along the nascent RNA and unwinds the RNA:DNA hybrid formed behind the transcribing complex to promote release of Pol II from DNA. This final step executes the full transcription termination process.

## Materials and methods

### Yeast strains

Strains used in this work are listed in the Supplemental Material. Temperature-sensitive mutants were grown in YPD at 25°C to OD_600_ = 0.4 and shifted for 1 h to 37°C unless indicated otherwise.

### Bioinformatics analyses

Available ChIP–chip data sets for Nrd1 and Pcf11 in wild-type cells and Pol II in *pcf11-9* versus wild type from Kim et al. (2010) were reanalyzed and compared with analysis of newly generated ChIP-seq data (accession no. GSE67483). More details regarding data analysis can be found in the Supplemental Material.

### ChIP and DIP

ChIP samples for qPCR and genome-wide analyses were prepared as described (Grzechnik and Kufel 2008). Precipitated DNA was amplified with Sensimix (Bioline) on a Rotogene (Corbett). Detailed protocol and quantification of ChIP/DIP values are described in the Supplemental Material. Detailed maps and amplicon locations for specific genes are presented in Supplemental Figure S5. For genome-wide analysis of *PCF11* mutants, chromatin was precipitated with CMA601 antibody (Stasevich et al. 2014). Deep sequencing was performed by the High-Throughput Genomics Group at the University of Oxford.

### Protein analyses

For immunoprecipitation experiments, protein extracts were incubated for 2 h at 4°C with protein A and G magnetic beads (Dynabeads, Life Technologies) preincubated with an appropriate antibody. For Western blot analysis, proteins were resolved in Tris-acetate gels (Life Technologies) and electrotransferred into nitrocellulose membrane. Working concentrations of particular antibodies are shown in the Supplemental Material.

## Supplementary Material

Supplemental Material
