# Supplementary material for: Pcf11 orchestrates transcription termination pathways in yeast
Source: Genes Dev. 2015 Apr 15;29(8):849–61. doi: 10.1101/gad.251470.114 (PMC4403260; doi:10.1101/gad.251470.114)
Supplement: Supplemental Material [file supp_29_8_849__index.html]

Supplemental Material 

# Pcf11 orchestrates transcription termination pathways in yeast

## Supplemental Material

**Files in this Data Supplement:**

- Supp Material.pdf
- Supp Table S5.xlsx
