# Supplementary material for: Pcf11 orchestrates transcription termination pathways in yeast
Source: Genes Dev. 2015 Apr 15;29(8):849–61. doi: 10.1101/gad.251470.114 (PMC4403260; doi:10.1101/gad.251470.114)
Supplement: Supplemental Material [file supp_29.8.849_SuppMaterial.pdf]

Supplemental Material

Supplemental Materials and Methods

Table S1. Strains used in this study

	name	genotype	reference
1.	WT for *sen1-1*	*MATα leu2-3,112 ura3-52 pep4-3*	(Ursic et al. 1997)
2.	*sen1-1*	*MATα leu2-3,112 ura3-52 pep4-3 sen1-1*	(Ursic et al. 1997)
3.	WT for *nrd1-102*	*MATα ura3Δ0 his3Δ1 leu2Δ0 met15Δ0*	(Conrad et al. 2000)
4.	*nrd1-102*	*MATα ura3Δ0 his3Δ1 leu2Δ0 met15Δ0 nrd1-102*	(Conrad et al. 2000)
5.	*nab3-11*	*MATa ade2 can1-100 his3-11,15 leu2-3,-1132 trp1-1 ura3-1 nab3-11*	(Conrad et al. 2000)
6.	*GAL1::SNR13*	as WT for *sen1-1* but *GAL1::kanMX6::SNR13*	This study
7.	*sen1-1 GAL1::SNR13*	as *sen1-1* but *GAL1::kanMX6::SNR13*	This study
8.	*nrd1-102 GAL1::SNR13*	as *nrd1-102* but *GAL1::kanMX6::SNR13*	This study
9.	WT for *sen1-1 NRD1-TAP*	as WT for *sen1-1* but *NRD1::TAP::URA3*	This study
10.	*sen1-1NRD1-TAP*	as *sen1-1* but *NRD1::TAP::URA3*	This study
11.	WT W303	*MATa ade2 his3 leu2 trp1 ura3*	Euroscarf
12.	*pcf11-13*	*MATa ura3-1 trp1Δ ade2-1 leu2-3, 112 his3-11, 15 pcf11Δ::TRP1 (pNOPL-pcf11-13)*	(Sadowski et al. 2003)
13.	*pcf11-2*	*MATa ura3-1 trp1Δ ade2-1 leu2-3, 112 his3-11,15 pcf11-2*	(Amrani et al. 1997)
14.	*pcf11-9*	*MATa ura3-1 trp1Δ ade2-1 leu2-3, 112 his3-11,15 pcf11-9*	(Amrani et al. 1997)
15.	*NRD1-TAP SEN1-HA*	as W303 but *NRD1::TAP::URA3 SEN1::6HA::kanMX6*	This study
16.	*pcf11-9 NRD1-TAP SEN1-HA*	as *pcf11-9* but *NRD1::TAP::URA3 SEN1::6HA::kanMX6*	This study
17.	*SEN1-HA*	as W303 but *SEN1::6HA::kanMX6*	This study
18.	*pcf11-9 SEN1-HA*	as *pcf11-9* but *SEN1::6HA::kanMX6*	This study
19.	W303 *NRD1-TAP*	as W303 but *NRD1::TAP::URA3*	This study
20.	*pcf11-13 NRD1-TAP*	as *pcf11-13* but *NRD1::TAP::URA3*	This study
21.	*rrp6Δ*	as W303 but *RRP6::HIS3*	J. Kufel, unpublished
22.	*rrp6Δ pcf11-13*	as *pcf11-13* but *RRP6::HIS3*	J. Kufel, unpublished
23.	*rrp6Δ pcf11-2*	*MATa ura3-1 trp1Δ ade2-1 leu2-3, 15 pcf11-2, RRP6::HIS*	J. Kufel, unpublished
24.	FD-4D (WT for *sen1-1*)	Matα *leu2 trp1Δ63 ura3*	(Rondon et al. 2009)
25.	FD-4B (*sen1-1*)	Matα *leu2 trp1Δ63 ura3 sen1-1*	(Rondon et al. 2009)
26.	*rrp6Δ* (FD-4D)	as FD-4D but *RRP6::KanMX6*	(Mischo et al. 2011)
27.	*sen1-1 rrp6 Δ*	as FD-4B but *RRP6::KanMX6*	(Mischo et al. 2011)
28.	*PCF11-HA*	as W303 but *PCF11-3HA::TRP1*	This study
29.	*GAL1::NRD1 PCF11-HA*	as *Pcf11-HA* but *GAL1::NRD1::KanMX6*	This study
30.	YLM127 (WT for *clp1-12*)	*clp1::TRP1 ade2-1 leu2-3,112 ura3-1 trp1Δ his3-11,15 can1-100 pHH2-CLP1*	(Haddad et al. 2011)
31.	YLM130 (*clp1-12*)	*clp1::TRP1 ade2-1 leu2-3,112 ura3-1 trp1Δ his3-11,15 can1-100 pHH2-clp1-12*	(Haddad et al. 2011)
32.	YLM127 *rrp6 Δ*	as YLM127 but *RRP6::HIS3*	This study
33.	*clp1-12 rrp6 Δ*	as YLM130 but *RRP6::HIS3*	This study

Table S2. Plasmids used in this study

	name	features	desription	reference
1.	pRS316	CEN *URA3*, amp	control for p*GAL1*::*PCF11*-CID	Euroscarf
2.	p*GAL1*::*PCF11*-CID	CEN *URA3*, amp	Plasmid expressing *PCF11* CID	This study
3.	pAG425GPD	2μ *LEU2*	Control for pAG425GPD-*PCF11*	(Honorine et al. 2010)
4.	pAG425GPD-*PCF11*	2μ *LEU2*	Plasmid overexpressing *PCF11*	(Honorine et al. 2010)
5.	pYM26	amp	Used for HA tagging	Euroscarf (Janke et al. 2004)
6.	pFA6a-*kanMX6*-p*GAL1*	amp	Used for *GAL1* insertion	(Longtine et al. 1998)
7.	pBS1539	amp	Used for TAP tagging	Euroscarf
8.	pUG*GAL1/10*	CEN *URA3*, amp	Backbone for p*GAL1*::*PCF11*-CID	(Tan-Wong et al. 2012)
9.	pKC173	CEN U*RA3*, amp *CUP1*::cMyc::*SEN1-R302W*	Used for Pol II co-immnoprecipitation with Sen1-R302W	(Chinchilla et al. 2012)

Table S3. Oligonucleotides used in this study

	name	Sequence 5'-3'	target
1.	5SNR13 IM	AATGGAGCCTGGTAAGTTCC	SNR13 (amp1)
2.	snr13 -1 R	ATATAGAGACGATTTGAGATGCC	SNR13 (amp1)
3.	2 SNR13-TRS F	CTGACCTTTTAACTTCCCCGTAG	SNR13 (amp2)
4.	snr13PE	ATGAACAAGCCAAACCCAAC	SNR13 (amp2)
5.	3 SNR13-TRS F	CACGGAAGCGACAGAAAGACAGGGAG	SNR13 (amp3)
6.	3 SNR13-TRS R	CTAGAGGGAATGTATGTTGTTGAAG	SNR13 (amp3)
7.	4 SNR13-TRS F	ATCACGGCGCCTCATCTTTG	SNR13 (amp4)
8.	4 SNR13-TRS R	GAGCATCTGCTTTCCTTTCAC	SNR13 (amp4)
9.	5 SNR13-TRS F	CGTTTCTTGTGAGTACTTCGTTTGCG	SNR13 (amp5)
10.	5 SNR13-TRS R	TCAGCCAAATCTCAAACCTTCCC	SNR13 (amp5)
11.	6 SNR13-TRS F	TCAGCCAAATCTCAAACCTTCCC	SNR13 (amp6)
12.	6 SNR13-TRS R	TAATTCAATATCAAAAACTTGGGAAGGC	SNR13 (amp6)
13.	1SNR5 F	TTTTCACAGCGCGCTCCTTG	SNR5 (amp1)
14.	1SNR5 R	GGAGGCGTGATGTCTTAAGCATG	SNR5 (amp1)
15.	2SNR5 F	GGTTCGCTCTAGGTGTACATATCTTCAC	SNR5 (amp2)
16.	2SNR5 R	AGCGCCTTAGCTGACTACAGCAC	SNR5 (amp2)
17.	3SNR5 F	AAATAGACCTTGCTCGAGAGATGTCTAG	SNR5 (amp3)
18.	3SNR5 R	TTACATGTGTATGTTTAATAAGCGGTACG	SNR5 (amp3) RT reverse primer
19.	4SNR5 F	GCCCTGCTACTGCTAACTTCATCAG	SNR5 (amp4)
20.	4SNR5 R	GATATCTCTTCTAATTTCACCCACGAG	SNR5 (amp4)
21.	5SNR5 F	GGTGACAGTAACCGCCTACCAGG	SNR5 (amp5)
22.	5SNR5 R	TGGCGCTGGTATAATTCAATAGCATC	SNR5 (amp5)
23.	6SNR5 F	TGCGTATACCCGCTACACCACC	SNR5 (amp6)
24.	6SNR5 R	CCAAAATCCAAGACCTATATGAGAGTTTGG	SNR5 (amp6)
25.	5-5UTR NRD1 F	TTACTAGCGCACGAGACAAG	NRD1 (amp1)
26.	5-5UTR NRD1 R	AGTAGCTCATTTCGACCATAAAC	NRD1 (amp1)
27.	5UTR Nrd1 F	CCTCGTTAGCATGACTCCTT	NRD1 (amp2)
28.	5UTR Nrd1 R	GGTTGTAGCAATGGATACGG	NRD1 (amp2)

29.	5Nrd1 F	GACTACTCAAGACTTTGTCCCG	NRD1 (amp3)
30.	5Nrd1 R	CAGTTCTTGAATTACTTCGCCC	NRD1 (amp3)
31.	MidNrd1 F	TTCCAAGTAATAGCGGCTCTG	NRD1 (amp4)
32.	MidNrd1 R	CGATGGCTGAGAAAATGGTG	NRD1 (amp4)<br>RT reverse primer
33.	3Nrd1 F	GGTCCAAGAGATTGTTGTGAC	NRD1 (amp5)
34.	3Nrd1 R	CCAACGATAATATCCGGTTCTTC	NRD1 (amp5)
35.	3UTR Nrd1 F	CCCACTGCTCAATTGAATTC	NRD1 (amp6)
36.	3UTRNrd1 R	GTTATGTGAATACATAAGGATGTCC	NRD1 (amp6)
37.	3UTR TAP F	AGCCAAAGCGCTAACCTTTTAG	NRD1 (amp6),<br>TAP-tagged strain
38.	3UTR TAP R	CTTAATGGTAAGGAACAACAAGCGG	NRD1 (amp6),<br>TAP-tagged strain
39.	1-1F PMA1	CCCTCGTTCACAGAAAGTCTGAAGAAG	PMA1 (amp1)
40.	1-1R PMA1	GGAGCATAAGCGGTACCCACC	PMA1 (amp1)
41.	2F PMA1	CCCCAGCTAGTTAAAGAAAATCATTGAAAAG	PMA1 (amp2)
42.	2R PMA1	CTTAGCAGGCTTTTCTTGAGTTGGC	PMA1 (amp2)
43.	3F PMA1	GTACGGTTTGAATCAAATGGCTG	PMA1 (amp3)
44.	3R PMA1	CACAGATAACACCGAAATCGACCC	PMA1 (amp3)
45.	4F PMA1	CGATCAATCTGCTATTACTGGTG	PMA1 (amp4)
46.	4R PMA1	CCAAAGCAGCAGCTCTACCAACGAAAG	PMA1 (amp4)<br>RT primer
47.	6F PMA1	CTTGGGTCTATGGATTGCTATTTTGG	PMA1 (amp5)
48.	6R PMA1	CTTGGTAGGTTCCATTTAACGGGCTTTGG	PMA1 (amp5)
49.	6.5F PMA1	TGGTGGTTTCTACTACGAAATGTCC	PMA1 (amp6)
50.	6.5R PMA1	TGATTAAATGCTACTTCAACAGGATTAGG	PMA1 (amp6)
51.	7F PMA1	GCCAACAAGAATAAGCCGCTTATTTCC	PMA1 (amp7)
52.	7R PMA1	CAAATAAAACAACCAGCTTCGGTGTGTG	PMA1 (amp7)
53.	9F PMA1	GCCTCCGCGAAATACCTTTACTGATTTTG	PMA1 (amp8)
54.	9R PMA1	AACTGAGTCATCTAGAGTAATGACGC	PMA1 (amp8)
55.	V2-1	GATATATGGGCTGGAGCGGTCC	chromosome V
56.	V2-2	ATAACCTTCGTTGCATTGACGGC	chromosome V
57.	5NrdTAPim	ACCATTGGCATCTCCTCCAC	NRD1 TAP-tagging
58.	3NrdTAPim	TTCATGGTACTGGATAAGCG	NRD1 TAP-tagging
59.	1TDH3 F	CACAACCTCAATGGAGTGATGC	TDH3 (amp1)
60.	1TDH3 R	GGGGAATAATTTCAGGGAACTGG	TDH3 (amp1)
61.	2TDH3 F	TTAGAGTTGCTATTAACGGTTTCGG	TDH3 (amp2)
62.	2TDH3 R	CATCGTGGGAAACTTCACCAGC	TDH3 (amp2)
63.	3TDH3 F	CTTGTACCACCAACTGTTTGGC	TDH3 (amp3)
64.	3TDH3 R	ATGGGATGATGTTACCGGAAGC	TDH3 (amp3)
65.	4TDH3 F	GAAGACGCTGTTGTCTCCTCTG	TDH3 (amp4)
66.	4TDH3 R	AAGCCTTGGCAACGTGTTCAAC	TDH3 (amp4)
67.	5TDH3 F	CTATTTTAATGACATTTTCGATTCATTG	TDH3 (amp5)
68.	5TDH3 R	CCAAAATTATTAAGAGCGCCTCC	TDH3 (amp5)
69.	6TDH3 F	GCCTATAAATCATGCCTATATTTGCG	TDH3 (amp6)
70.	6TDH3 R	CCGCGGGAATCTGTGTATATTAC	TDH3 (amp6)
71.	5GLSNR13	CTTTACATATAAAAGGGAAGGATTTTGAAATTATAAATGG<br>CATCTCAAATGAATTCGAGCTCGTTTAAAC	Construction of<br>*GAL1::SNR13*
72.	3GAL132s	CTCGAGCCAAATGCACTCATATTCATCATATAAAAAGGAA<br>AAAACTTCCTACTTTTATTACATTTGAATA	Construction of<br>*GAL1::SNR13*
73.	5GAL Nrd1	GTATTGTAGTTTTCTCTGATTACACATTTTTTCTAGTACT	Construction of

		TTTCTCCAAGGAATTCGAGCTCGTTTAAAC	*GAL1::NRD1*
74.	3GAL Nrd1	AATGATTCCAAGGTAGCTACAAAATTTTGAAAATCGTCGT CCTGCTGCATCATTTTGAGATCCGGGTTTT	Construction of *GAL1::NRD1*
75.	5Nel025c	AATTATGTTCCTATAGGAAG	*NEL025c* riboprobe
76.	Nel025c T7 probe	TAATACGACTCACTATAGGGAGAGTATCGAAATGATTGTT GGCG	*NEL025*c riboprobe
77.	F PGK1	TTGCGTTACCACATCGAAGAAGAAG	*PGK1* riboprobe
78.	R PGK1 T7	TAATACGACTCACTATAGGGAGACAATGATGATAGAGTCG ACCTTGTC	*PGK1* riboprobe
79.	SCR1 F	CCTTCCTCGCGGCTAGACACGG	*SCR1* riboprobe
80.	SCR1 R T7	TAATACGACTCACTATAGGGAGACACAATGTGCGAGTAAA TCCTGATG	*SCR1* riboprobe
81.	SNR13-TRS31	GTTGAATTATTCTTTGAGAC	RT primer
82.	5 PCF11 6HA	TCTAATAGTGGCAAGGTCGGTTTGGATGACTTAAAGAAAT TGGTCACAAACGTACGCTGCAGGTCGAC	*PCF11*-HA tagging
83.	3 PCF11 6HA	ATAATATATAGTTATTAAATTTAAATGTATATATGCAGTT CTGCTCTTATATCGATGAATTCGAGCTCG	*PCF11*-HA tagging
84.	5PCF11 klon	ATCCCCGGGATGGATCACGACACAGAAGTTATAG	*PCF11* CID cloning
85.	3Pcf CID NLS1	CTGGTGCACCAAAATCGCGCCTTCTCTTTTTGCTTAACATT GCTTGCAGGTTTTTCTGGTGGAG	*PCF11* CID cloning
86.	3Pcf CID NLS2	TAAGTCGACTCATAGACCTCTTCTTGGCCTTTTATTCGCTG GTGCACCAAAATCGCG	*PCF11* CID cloning
87.	5 SEN1 6HA	GGAATGCTTCATCTAGCCCATTTATCCCAAAAAAAAGAAA GCCTAGATCACGTACGCTGCAGGTCGAC	*SEN1*-HA tagging
88.	3 SEN1 6HA	GTATACACCAATATATATGCAGGTATAATTCCTAACACTT TTACTTCAAGATCA ATCGATGAATTCGAGCTCG	*SEN1*-HA tagging

Table S4. Antibodies used for protein analyses

	Name/ target	Reference/Source	Concentration used in WB analyses
1.	Anti-Nrd1	(Steinmetz and Brow 1998) from J. Corden	1:5000
2.	2F12 (anti-Nab3)	(Wilson et al. 1994) from J. Corden	1:250
3.	Ab9101 Anti-HA	Abcam	1:5000
4.	Anti-Tub2	Santa Cruz	1:500
5.	PAP (peroxidase-anti-peroxidase) (binds protein A)	Sigma	1:5000
6.	y-80 (Anti N-terminus of Rpb1)	Santa Cruz	1:250
7.	Ab5095 Anti-phospho S2 (anti-CTD phosphorylated at Ser2)	Abcam	1:2000
8.	Ab5131 Anti-phospho S5 (anti-CTD phosphorylated at Ser5)	Abcam	1:2000
9.	Anti-Ctk1	(Cho et al. 2001) from J Greenblatt	1:1000
10.	Anti-Fcp1	(Cho et al. 2001) from J. Greenblatt	1:1000
11.	Ab9132 Anti-Myc	Abcam	1:10000
12.	CMA601 (Anti-CTD)	(Stasevich et al. 2014)	1:5000

13.	Anti-Rabbit	Sigma	1:25000
14.	Anti-Mouse	Sigma	1:25000
15.	Anti-Goat	Sigma	1:25000

Table S5.

Separate excel file.

Strains growth conditions

Strains were grown in YPD (1% yeast extract, 2% Bacto-peptone, 2% glucose) with the following exceptions: i) cells expressing *PCF11*-CID were pre-grown in YP 2% raffinose in 25°C and transferred to YPGAL (1% yeast extract, 2% Bacto-peptone, 2% galactose) for 2 hr prior to 1 hr shift to non-permissive temperature (37°C); ii) For PCF11 overexpression, strains were grown in synthetic medium lacking uracil (0.67% yeast nitrogen base, 2% glucose, supplemented with respective amino acids and nucleotide bases); iii) cells used in analysis of Pcf11 recruitment were grown on YPGAL and shifted to YPD for indicated time.

Ts strains and respective isogenic WT were grown at 25°C until OD_{600} reached 0.4, diluted 2x with pre-warmed to 42°C medium and shifted to 37°C for 1 hr.

Strains subjected to growth tests were grown on liquid medium to OD_{600}=0.35. These were then harvested, washed and diluted in ddH_2O. 10-fold dilutions were spotted on the plates. Strains used in *PCF11*-CID complementation were grown on liquid synthetic medium in the absence of uracil (-ura) with 2% raffinose as a carbon source and plated on synthetic –ura medium contained either 2% galactose and 2% raffinose or 2% glucose. Strains

used in analysis of relation between Rrp6 and Pcf11 were pre-grown and plated on SD medium at indicated temperature.

Strains and plasmids construction

The transformation procedure was as described (Gietz et al. 1992). Strains were generated using one-step PCR method (Longtine et al. 1998). To obtained W303 *NRD1-TAP,* TAP cassette was amplified from pBS1539 plasmid (Euroscarf). The other *NRD1-TAP* strains was constructed using PCR product containing TAP cassette amplified from genomic DNA of W303 *NRD1::TAP*. *GAL1* ptomoter was amplified on pFA6a-kanMX6-PGAL1 template (Longtine et al., 1998) while 6HA tag on pYM26 (Euroscarf) (Janke et al. 2004).

Pcf11 CID was amplified from genomic DNA using primers 93 and 94 (see Supplementary Table). SMD1 NLS was added when the PCR product was re-amplified with primers 93 and 95. XmaI and SalI restriction sites were added at overhangs. The product was cleaved and ligated into pUGGAL1/10 (alias pUGCYC1) (Tan-Wong et al. 2012) in the place of *CYC1* gene.

RNA methods

Total RNA was isolated using a hot phenol procedure (Schmitt et al. 1990).

RNA (8μg) was separated on a 1% or 1.5% denaturing formaldehyde MOPS agarose gel and transferred to nylon membranes using a pressure blotter. Hybridizations were performed at 68°C with RNA in PerfectHyb buffer (Sigma) for at least 6 hr. Primers for probe templates are listed in Supplementary Table. α^{32}P UTP labeled riboprobes were made by *in vitro* transcription using MaxiScript (Ambion). Blots were washed once with 2xSSC 0.1% SDS and twice in

0.5xSSC 0.1% SDS at 68°C. Images were visualized by FLA5000 scanner (Fuji) and analyzed by AIDA Advanced Image Analyzer software (Raytest).

Transcriptional shut-off experiment

Cells were grown on YPD medium to OD_{600} 0.4. Next cultures were mixed with equal volume of YPD pre-warmed to 42°C and incubated for 30 min at 37°C. Cells were briefly centrifuged and concentrated in 10 ml of pre-warmed to 37°C YPD. Next thiolutin was added at 10μg/ml. 1 ml samples taken in the following time points were rapidly chilled in ice bath, washed with ice-cold water and frozen.

Transcriptional pulse-stop experiment

Cells were grown at 25°C on synthetic medium supplemented with 2% raffinose to OD_{600} 0.2. To induce *GAL1:SNR13* transcription, galactose was added to final concentration 2%. After 30 min cultures were mixed with an equal volume of pre-warmed to 42°C SC 2% raffinose 2% galactose medium and shifted to 37°C for 2 hr. To stop transcription glucose was added to concentration 4%. Samples collected at indicated time points following glucose repression and were subjected to ChIP analysis as described below.

Chromatin imunnoprecipitation (ChIP and DIP)

Formaldehyde was added to 1% and cultures were incubated with gently rotation for 5, 10 or 15 min (for RNA:DNA hybrids, Nrd1-TAP and Pol II precipitation respectively) at room temperature. Next glycine was added to quench formaldehyde. Cells were washed with PBS and frozen in -80°C. To

prepare the extracts pellets were resuspended in 1 ml ice cold FA1 and 500 μl of ice-cold glass beads were added. Cells were broken in MagnaLyser (set for 30s 7000 rpm, 2 cycles with incubation on ice in between). Extracts were sonicated in Bioruptor (Diagenode) in 15 ml tubes with the metal tip inside for 15 min (30 s on, 30 s off) on medium power. Samples were centrifuged in 1.5 ml tubes for 30 min at 14000 rpm. For one IP 1/3 of recovered supernatant in a volume of 700 μl was used. 5 μg of S9.6 or 2 μg of either 8WG16 (Covance), Anti-phospho S5 (ab5131, Abcam), Anti-phospho S2 (ab5095, Abcam) or Anti-HA (Abcam) antibody were incubated overnight at 4°C. Protein A agarose (Milipore) was used to precipitate S9.6, Anti-phospho S5 and Anti-phospho S2 while protein A magnetic beads (Dynabeads, Life Technologies) for Anti-HA. For 8WG16 Protein G agarose (Milipore) was employed. In the case of S9.6, 100 ng of salmon/herring sperm DNA was also added to incubation. To precipitate Nrd1-TAP, IgG Sepharose 6 Fast Flow (GE Healthcare) was used. Samples were washed as described (Grzechnik and Kufel 2008). Beads were suspended in 195 μl elution buffer with 5 μl Proteinase K and incubated for 1 hr at 42°C. To reverse the crosslink samples were incubated at 65°C for 4 hr and DNA was purified by Qiagen PCR purification kit. Final elution was in 50 μl. INPUT (20 μl from 700 μl used for IP) was diluted 25x prior to qPCR analyses. Reactions were run in a Rotogene (Corbett) using Sensimix (Bioline) kit. Relative enrichments were were quantified using the formula $2^{\Delta\Delta Ct} = 2^{((Ct\ INPUT\ control - Ct\ IP\ control) - (Ct\ INPUT\ target\ gene - Ct\ IP\ target\ gene))}$, where "Ct IP" and "Ct Input target gene" are cycle numbers for the gene of interest and "Ct IP" and "Ct Input control" are cycle numbers for non-coding

region on chromosome V. Detailed position and chromosomal features of analyzed transcription units are shown in Figure S5.

Protein analyses

Yeast were broken in lysis buffer (30 mM HEPES, pH 7.5, 200 mM KOAC, 1 mM MgOAC, 1 mM EGTA, pH 8.0, 10% glycerol, 0.05% Tween 20 supplemented with Complete protease inhibitor cocktail and phosphatase inhibitor cocktail (Roche) by vortexing 5 times for 30 sec with zirconia beads. For immunoprecipitation experiments, protein extracts were incubated for 2 hr at $4^{O}C$ with protein A and G magnetic beads (Dynabeads, Life Technologies) pre-incubated with an appropriate antibody. Beads were washed 5 times with 1.5ml of lysis buffer and suspended in 0.5% SDS. Bounded proteins were eluted by heating at $70^{O}C$ for 10 min. For Western Blot analysis, proteins were resolved in Tris-acetate gels (Life Technologies), electrotransferred into nitrocellulose membrane and incubated with antibodies. Working concentrations of particular antibodies are shown in Table S4 "Antibodies used for protein analyses".

Bioinformatics analyses

Data: For genomic analysis we used published CUTs, SUTs and XUTs coordinates (Xu et al. 2009; van Dijk et al. 2011). Genomic coordinates of TSS from (Nagalakshmi et al. 2008) were used for all genes if available. For genes where they were not determined in this dataset, TSS from either (Yassour et al. 2009) or (Zhang and Dietrich 2005) were used. Genomic coordinates for NUTs (Schulz et al. 2013) were obtained from the authors. List of NRD-dependent protein-coding genes from (Schulz et al. 2013) was complemented with data from (Arigo

et al. 2006; Steinmetz et al. 2006; Kuehner and Brow 2008; Thiebaut et al. 2008; Creamer et al. 2011). All genomic coordinates were mapped to UCSC sacCer3 (genome version V64, February 2011) and apart from NUTs, they were all downloaded from the SGD (Saccharomyces Genome Database) (Cherry et al. 2012). ChIP-chip data (log2 ratios of ChIP signal/input) for Nrd1, Pcf11 and Pol II in WT and *pcf11-9* cultured at 23^{0}C and 37^{0}C have been published (Kim et al. 2010). Newly generated ChIP-sequencing data sets after read count normalization were further processed in a similar way to the ChIP-chip data: after creating genome coverage files the signal was binned into 20 bp intervals for both IP and input files. Subsequently a constant of 1 was added to all bins and then log2 form the Ip/input value was calculated.

Binding profiles of Nrd1, Pcf11 and Pol II for NRD-dependent protein-coding genes, snoRNA, CUTs, SUTs and XUTs were generated, based on above mentioned genomic coordinates and ChIP-chip data, in the R environment (RCoreTeam 2014). Small fraction of genes with signal discontinuities was excluded from analyses. Binding profiles for snoRNA were aligned to their 3' mature ends; binding profiles of CUTs, SUTs, XUTs and protein-coding genes were aligned to their TSS. To generate averaged binding profiles of Nrd1 and Pcf11 all transcription units in particular groups were used. In case of Pol II signal only genes fulfilling the conditions described below were qualified. Both, single loci profiles and averaged profiles signals, were smoothed by a moving average with 220 nt window responding to the average length of the sonicated chromatin fragments from ChIP experiments.

Heat maps: Matrices for heat maps were prepared based on ChIP-chip signal from (Kim et al. 2010). For all loci signal values for analysed objects sequence, together with 500 nt upstream of TSS (mature 5' end for snoRNA) and 1000 nt downstream of the 3' end was extracted. For snoRNA, signals were aligned to the mature 3' end and to TSS for protein-coding genes, CUTs, SUTS and XUTs. To define TSS (or snoRNA mature 5' ends) and 3' ends three light grey pixels were inserted responding to zero value from the color scale. Empty spaces were filled with zeros and matrices generated in such way were visualized using the ggplot2 package (Wickham 2009) in the R environment.

Termination defect analysis: To evaluate Pcf11 transcription termination dependency, RNA Pol II signals in WT, *pcf11-9* (Kim et al. 2010), *pcf11-2*, *pcf11-9* and *pcf11-13* were compared. As reference points we used mature 3' ends for snoRNA and TSS for CUTs, SUTs, XUTs and protein-coding genes (point A; Figure S1). Transcription units of interest where the average RNA Pol II log2 (ChIP/input) signal value in the 60 nt region covering the reference point was above local threshold were subject for further analysis. Protein-coding genes and snoRNA were considered as Pcf11-dependent if they fulfilled at least one of two conditions: 1) the average RNA Pol II signal from a 60 nt region covering the control point in at least 1 of 3 control points (point B; Figure S1) located 300, 600 and 900 nt downstream reference point decreased in WT by more than 40%, while the responding signal in *pcf11-9* either increased or did not decrease by more than 30%; 2) the average signal of 200 nt downstream the reference point in *pcf11-9* was above the threshold of 0.4 and was at least 2 times higher than respective average signal in WT. For CUTs, SUTs and XUTs this algorithm was

slightly modified – the control points were placed every 300 nt till gene end. Known examples of NRD-dependent protein-coding genes *HRP1*, *ADE12* and *SRG1-SER3* were not qualified for analysis therefore their mRNA accumulation was assessed in *pcf11-13* by Northern Blot (data not shown). Based on this, *HRP1*, *ADE12* and *SRG1-SER3* attenuation was classified as Pcf11-dependent. Transcription termination of particular loci was considered as Nrd1-dependent if reference point +/- 250 nt overlapped with 5' termini of Nrd1-unterminated transcripts (NUTs).

Statistical analysis: Significance of differences of average signal peak localizations was estimated using t test with Bonferroni correction where applicable. Comparisons between ratios of transcription units dependent on distinct termination pathways in different units groups was assessed with Chi square test for categorical data.

Supplemental Figure Legends

Figure S1. Principals of bioinformatics analyses of the termination defect in *PCF11* mutants

Figure S2. Pol II distribution in WT and *pcf11-9* over investigated ncRNA loci

Heat maps showing Pol II ChIP-chip signal from 0.5kb upstream of TSS and 1kb downstream of mature 3' end for CUTs (A), SUTs (B), XUTs (C) and NAPC genes (D) in WT and *pcf11-9*. Signals are aligned relative to TSS (vertical white lines marked with red arrows). 3' ends of transcription units are marked by spacers specific to each gene.

Figure S3. Metagene analysis of SUTs and XUTs in *PCF11* mutants

Pol II average distribution 0.5kb upstream and 1.5kb downstream relative to SUTs and XUTs TSS (X axis) is shown. ChIP-seq signals smoothed with 220 nt moving average.

Figure S4. Nrd1 and Pcf11 recruitment over NRD-attenuated (NAPC) and NRD-independent protein-coding genes

Metagene analysis of Nrd1 and Pcf11 localization based on ChIP-chip data bases (Kim et al 2010) across protein coding genes, either NRD-dependent (NAPC) or independent. ChIP-chip signals smoothed with 220 nt moving average aligned to TSS.

Figure S5. Detailed diagrams of analyzed genes

Arrows denote TSS and direction of transcription while PAS poly(A) site. Blue rhomboids show position of functional NBS. The region of potential NRD-termination is marked by red. Red intensity indicates possibility of termination event. Positions of amplicons are shown below each diagram.

Figure S6. Nrd1 and Pcf11 recruitment over NRD-dependent genes

(A) Northern Blot showing levels of *PCF11* mRNA in analyzed cells. Five times less of total RNA isolated from the strain overexpressing *PCF11* was loaded on the gel. SCR1 and rRNA are shown as loading controls. (B) Signal ratio originating from amplicon 3 and 2 in WT and *pcf11-13*. (C1) ChIP analysis of Nrd1-TAP recruitment in *sen1-1*. Diagrams, growth condition and ChIP quantification as described for Fig. 4. Error bars represent standard deviations. Trend lines emphasize Nrd1 profiles. (C2) The ratio of signal from amplicon 3 to amplicon 2 in *sen1-1.*

Figure S7. Analysis of genetic interactions of CFIA mutant with Rrp6

(A) Growth curves for *rrp6Δ/pcf11-13* and *rrp6Δ/pcf11-2* at 25°C and 37°C . Cells were grown on YPD. For analysis at non-permissive temperature, incubation for 30 min at 37°C was followed by first measurement. Doubling time was calculated using http://www.doubling-time.com/compute.php (Roth V. 2006). (B) Growth tests of *rrp6Δ/pcf11-13* (W303 background) and *rrp6Δ/sen1-1* (FD4D background) under permissive conditions. 1:10 dilutions grown at 25°C in SD complete medium for 3 days. (C) Growth curves for *rrp6Δ/clp1-12*. Cells were grown in YPD. For analysis at 32°C and 37°C, cells were shifted from 25°C to

respective temperature for 30 min prior to first measurement. (D) Calculation of the *PGK1* mRNA half-lives (t1/2) in *pcf11-13* and *sen1-1*. The best-fit lines were determined for plotted quantifications of the Northern Blot analysis from the Fig. 4E normalized to the loading control.

Figure S8. Analysis of CTD Ser2 phosphorylation in *pcf11-13* mutant

A) Ser2-P CTD levels (not normalized to total Pol II levels) over *SNR13-TRS31*, *SNR5-HEM4* and *NRD1*. Total Pol II (B) and Ser2-P CTD (not normalized to total Pol II levels) (C) over *PMA1* and *TDH3*. Diagrams, growth condition and ChIP quantification are as described for Figure 4. Error bars represent standard deviations. D) PonceauS staining of the membrane used for Ser2-P detection in Figure 5E.

Figure S9. Pol II kinetics in WT, *sen1-1* and *nrd1-102* at *GAL1::SNR13-TRS31.*

Not normalized ChIP Pol II values used for analyses shown in Figure 7D. Time points 0, 1 and 15 min on glucose are shown.

Fig. S1

General principals of transcription read-through analysis

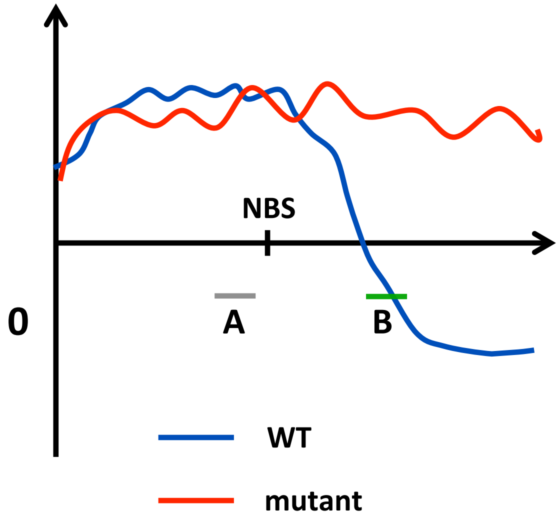

Transcription termination failed if at least one of two conditions was fulfilled:

1. The average Pol II signal from a 60 nt region covering the control point (B) in at least 1 of 3 control points located 300, 600 and 900 nt downstream reference point (A) decreased in WT by more than 40%, while the responding signal in *pcf11-9* mutant strain either increased or did not decrease by more than 30%:

$$B_{(WT)}/A_{(WT)} < 0.6 \quad \text{and} \quad B_{(MT)}/A_{(MT)} > 0.7$$

2. The average signal of 200 nt downstream the reference point in *pcf11-9* mutant strain ($\bar{A}_{200(MT)}$) was above the threshold of 0.4 and was at least 2 times higher than respective average signal in WT ($\bar{A}_{200(MT)}$). For CUTs, SUTs and XUTs this algorithm was slightly modified – the control points were placed every 300 nt till the end of the gene.

$$\bar{A}_{200(MT)} > 0.4 \quad \text{and} \quad \bar{A}_{200(MT)} / \bar{A}_{200(WT)} > 2$$

Fig. S2

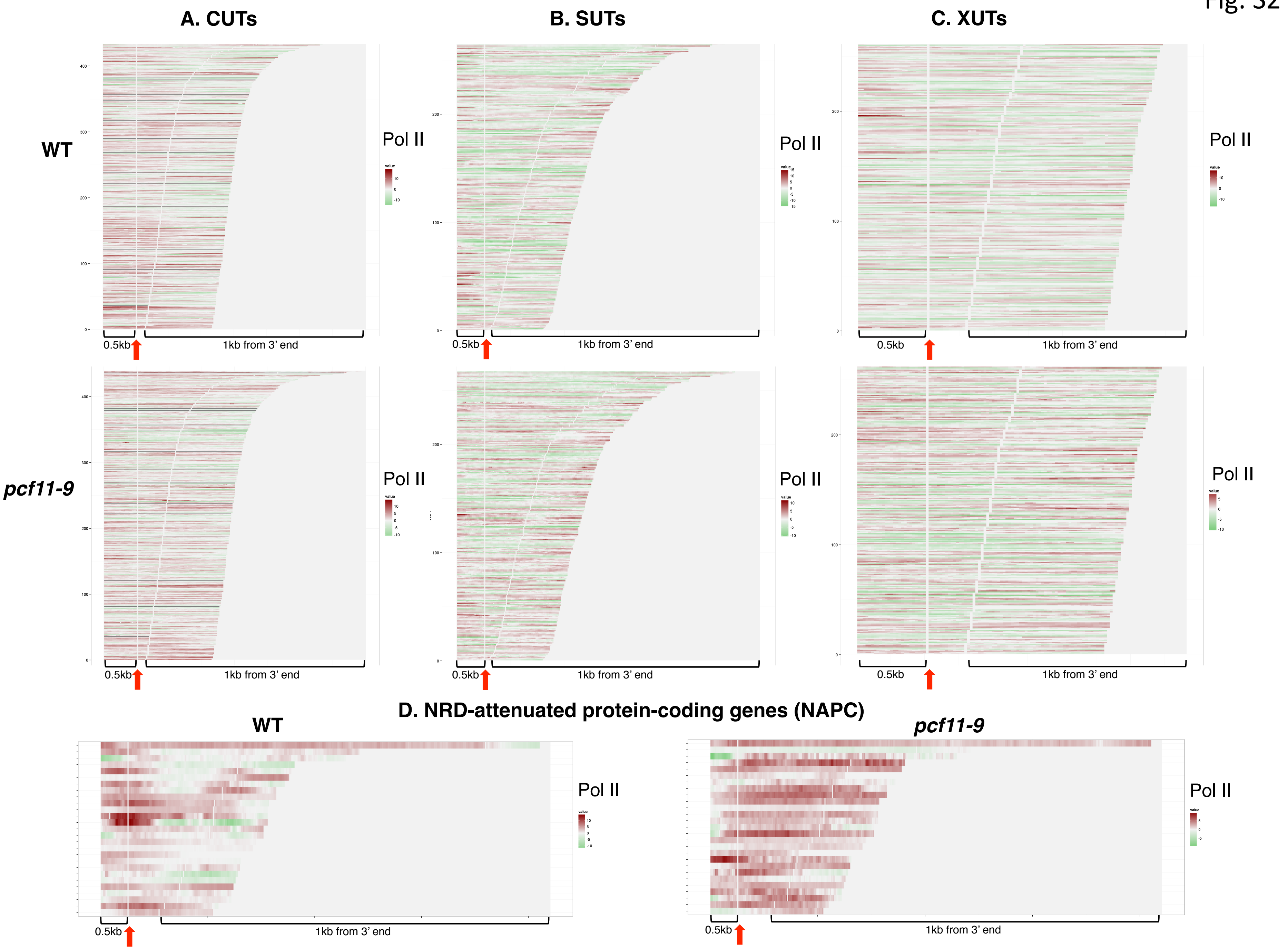
A. CUTs
B. SUTs
C. XUTs
WT
pcf11-9
Pol II
0.5kb
1kb from 3' end
D. NRD-attenuated protein-coding genes (NAPC)
WT
pcf11-9

Fig. S3

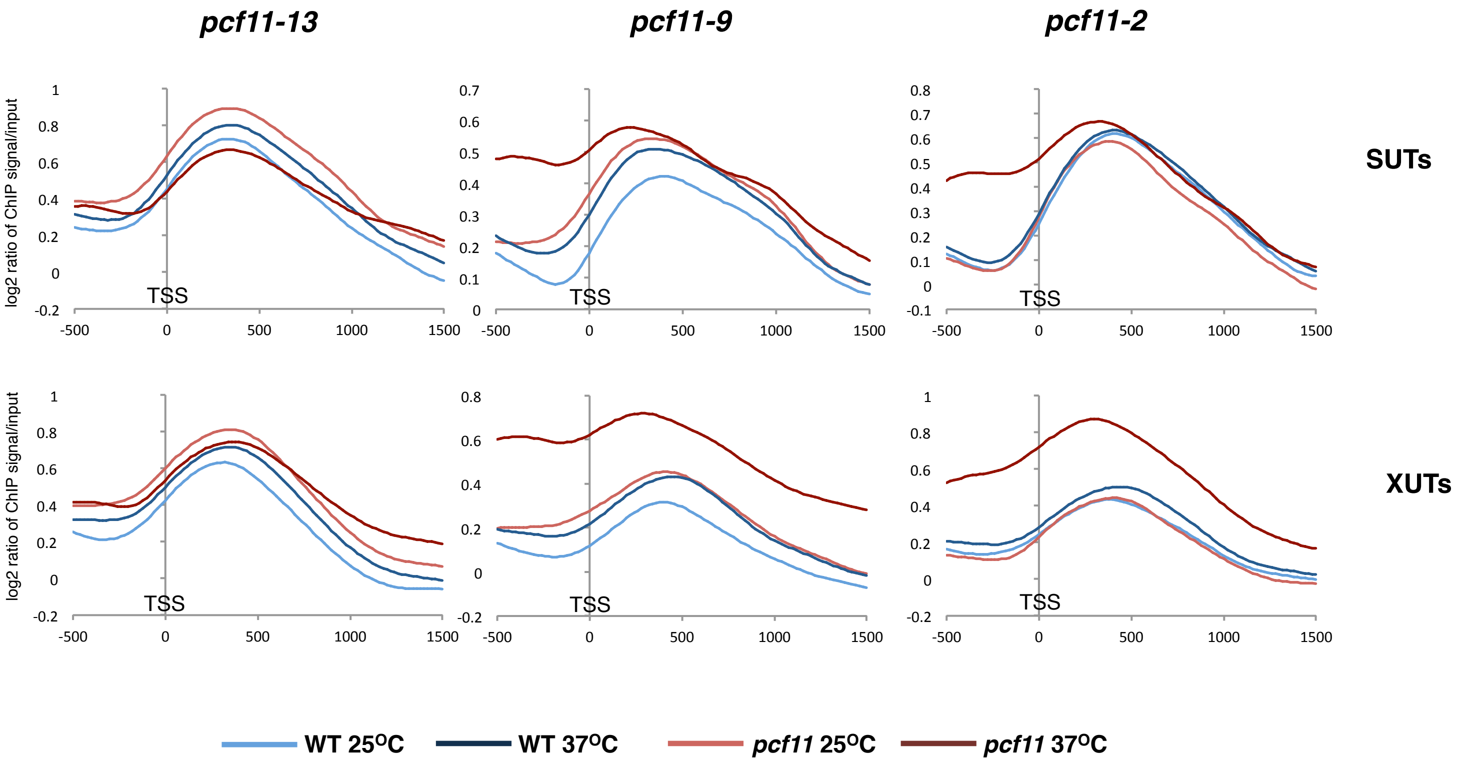

pcf11-13
pcf11-9
pcf11-2
SUTs
XUTs
log2 ratio of ChIP signal/input
TSS
-500
0
500
1000
1500
WT 25°C
WT 37°C
pcf11 25°C
pcf11 37°C

Fig. S4

protein-coding genes

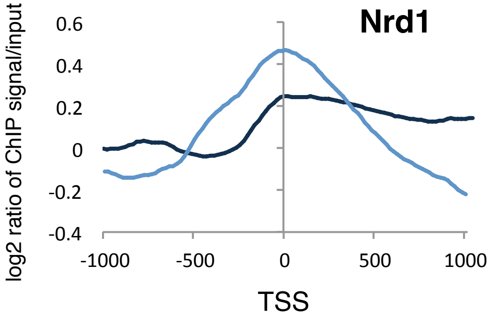

NRD-attenuated protein-coding (NAPC) genes
NRD-independent (40 randomly selected active genes)

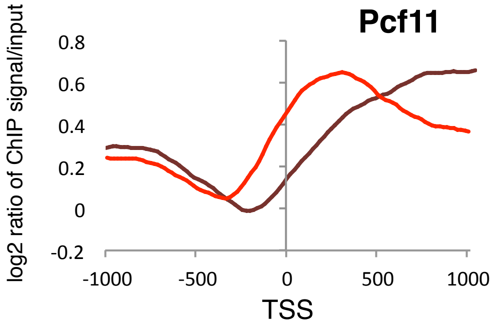

NRD-attenuated protein-coding (NAPC) genes
NRD-independent (40 randomly selected active genes)

Fig. S5

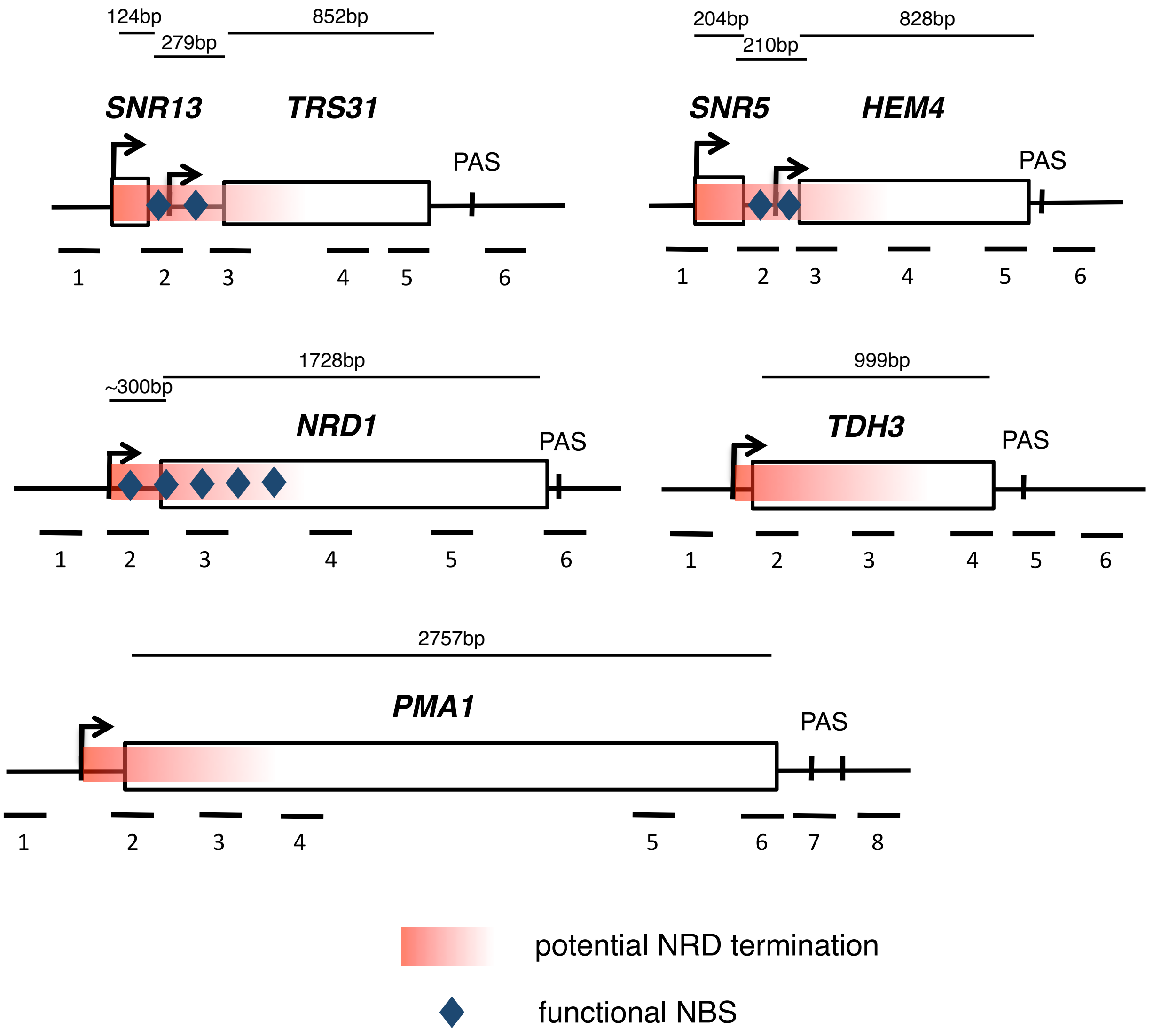
124bp
279bp
852bp
SNR13
TRS31
PAS
1 2 3 4 5 6
204bp
210bp
828bp
SNR5
HEM4
PAS
1 2 3 4 5 6
1728bp
~300bp
NRD1
PAS
1 2 3 4 5 6
999bp
TDH3
PAS
1 2 3 4 5 6
2757bp
PMA1
PAS
1 2 3 4 5 6 7 8
potential NRD termination
functional NBS

Fig. S6

A.

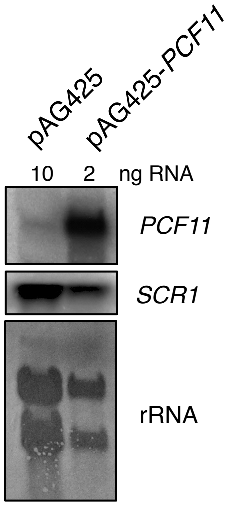

B.

Ratio of amplicon 3:2

	WT	*pcf11-13*
SNR13	0.97	1.74
SNR5	0.43	1.64
NRD1	1.01	2.55

C1.

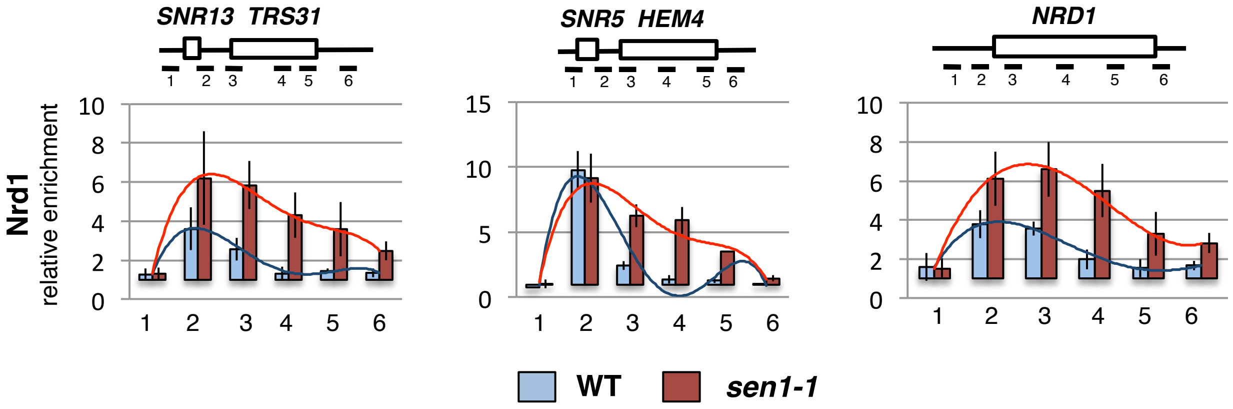

C2.

Ratio of amplicon 3:2

	WT	*sen1-1*
SNR13	0.7	0.94
SNR5	0.35	0.57
NRD1	0.93	0.99

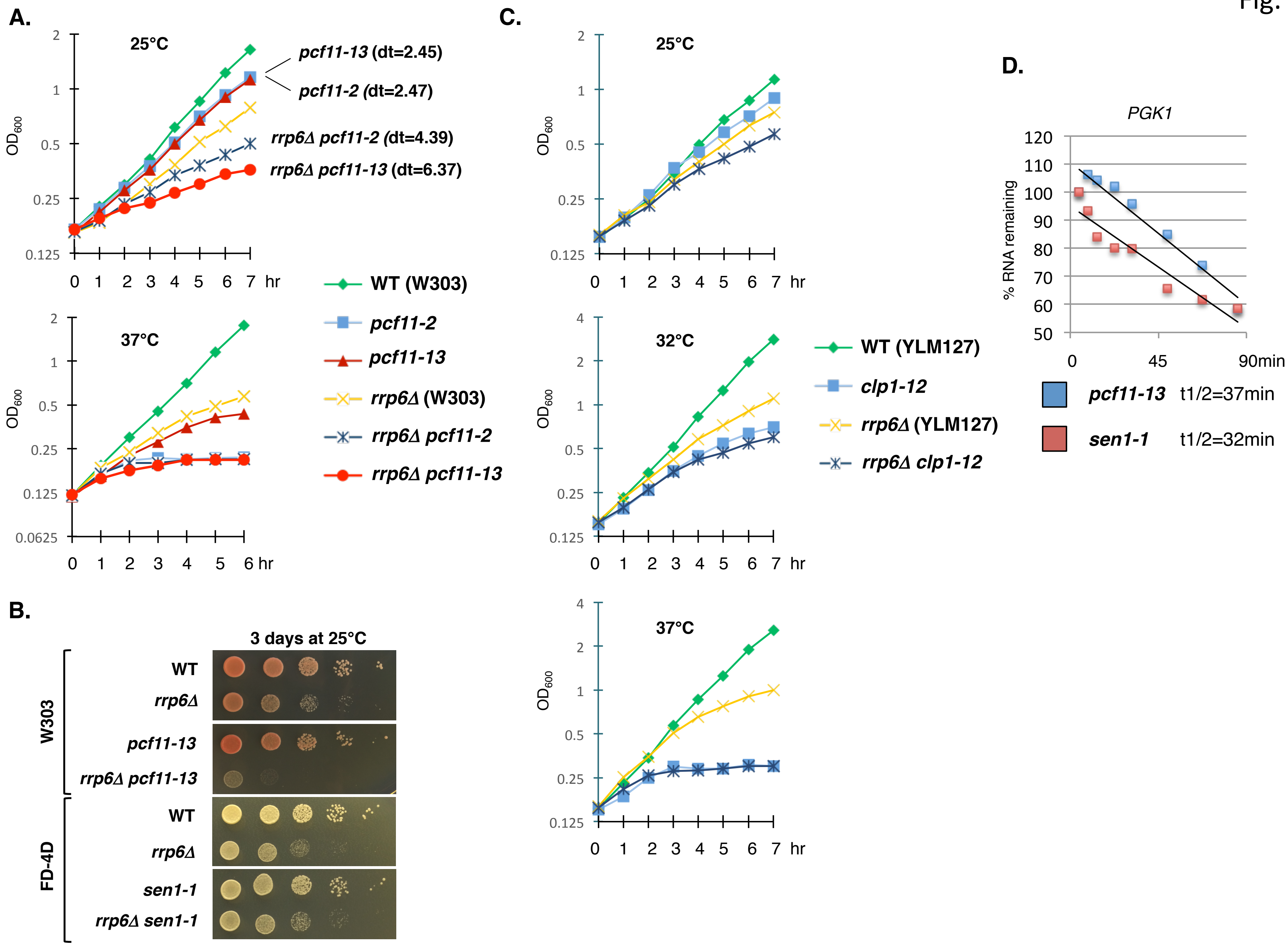
A.
25°C
pcf11-13 (dt=2.45)
pcf11-2 (dt=2.47)
rrp6Δ pcf11-2 (dt=4.39)
rrp6Δ pcf11-13 (dt=6.37)
OD600
37°C
WT (W303)
pcf11-2
pcf11-13
rrp6Δ (W303)
rrp6Δ pcf11-2
rrp6Δ pcf11-13
hr
B.
3 days at 25°C
W303
WT
rrp6Δ
pcf11-13
rrp6Δ pcf11-13
FD-4D
WT
rrp6Δ
sen1-1
rrp6Δ sen1-1
C.
25°C
32°C
37°C
WT (YLM127)
clp1-12
rrp6Δ (YLM127)
rrp6Δ clp1-12
D.
PGK1
% RNA remaining
90min
pcf11-13 t1/2=37min
sen1-1 t1/2=32min

Fig. S8

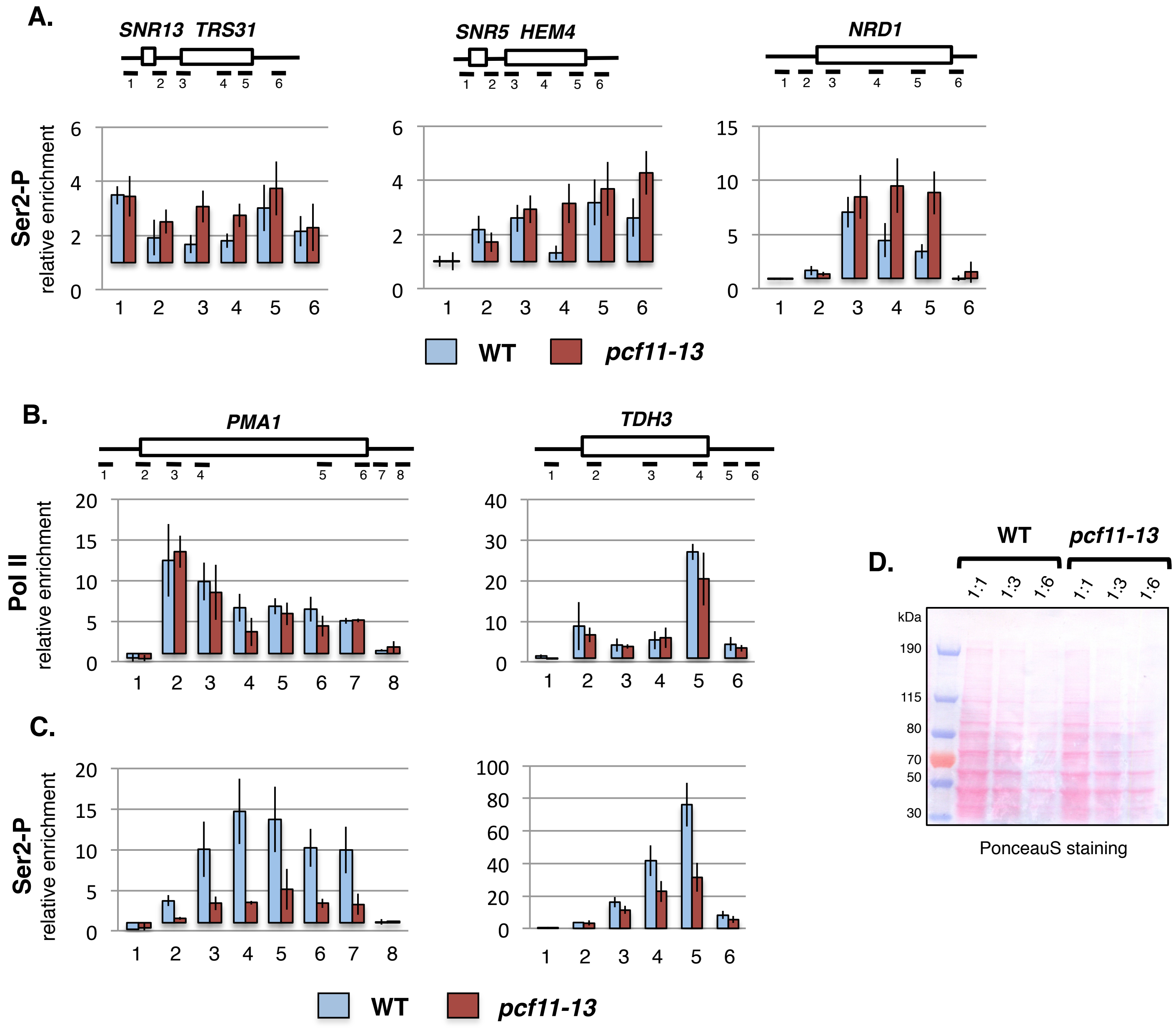
A.
SNR13 TRS31
SNR5 HEM4
NRD1
Ser2-P
relative enrichment
WT
pcf11-13
B.
PMA1
TDH3
Pol II
relative enrichment
C.
Ser2-P
relative enrichment
WT
pcf11-13
D.
WT
pcf11-13
1:1
1:3
1:6
kDa
190
115
80
70
50
30
PonceauS staining

Fig. S9

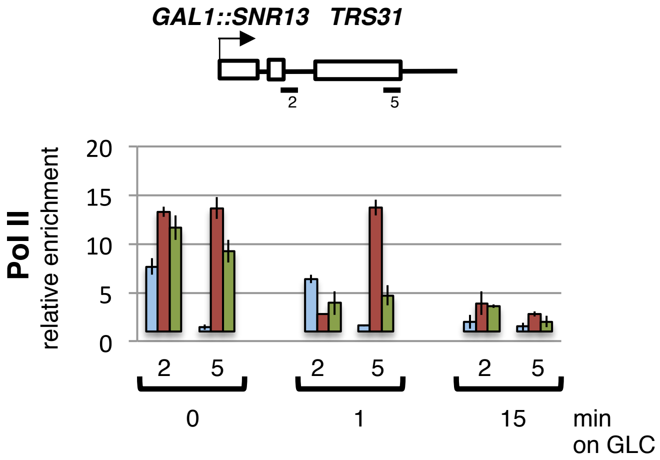

GAL1::SNR13 TRS31
2
5
Pol II
relative enrichment
20
15
10
5
0
2 5 2 5 2 5
0
1
15
min
on GLC

Supplemental Literature

Amrani N, Minet M, Wyers F, Dufour ME, Aggerbeck LP, Lacroute F. 1997. PCF11 encodes a third protein component of yeast cleavage and polyadenylation factor I. *Mol Cell Biol* **17**: 1102-1109.

Arigo JT, Carroll KL, Ames JM, Corden JL. 2006. Regulation of yeast NRD1 expression by premature transcription termination. *Mol Cell* **21**: 641-651.

Cherry JM, Hong EL, Amundsen C, Balakrishnan R, Binkley G, Chan ET, Christie KR, Costanzo MC, Dwight SS, Engel SR et al. 2012. Saccharomyces Genome Database: the genomics resource of budding yeast. *Nucleic Acids Res* **40**: D700-705.

Chinchilla K, Rodriguez-Molina JB, Ursic D, Finkel JS, Ansari AZ, Culbertson MR. 2012. Interactions of Sen1, Nrd1, and Nab3 with multiple phosphorylated forms of the Rpb1 C-terminal domain in Saccharomyces cerevisiae. *Eukaryot Cell* **11**: 417-429.

Cho EJ, Kobor MS, Kim M, Greenblatt J, Buratowski S. 2001. Opposing effects of Ctk1 kinase and Fcp1 phosphatase at Ser 2 of the RNA polymerase II C-terminal domain. *Genes Dev* **15**: 3319-3329.

Conrad NK, Wilson SM, Steinmetz EJ, Patturajan M, Brow DA, Swanson MS, Corden JL. 2000. A yeast heterogeneous nuclear ribonucleoprotein complex associated with RNA polymerase II. *Genetics* **154**: 557-571.

Creamer TJ, Darby MM, Jamonnak N, Schaughency P, Hao H, Wheelan SJ, Corden JL. 2011. Transcriptome-wide binding sites for components of the Saccharomyces cerevisiae non-poly(A) termination pathway: Nrd1, Nab3, and Sen1. *PLoS Genet* **7**: e1002329.

Gietz D, St Jean A, Woods RA, Schiestl RH. 1992. Improved method for high efficient transformation of intact yeast cells. *Nucleic Acids Res* **20**: 1425.

Grzechnik P, Kufel J. 2008. Polyadenylation linked to transcription termination directs the processing of snoRNA precursors in yeast. *Mol Cell* **32**: 247-258.

Haddad R, Maurice F, Viphakone N, Voisinet-Hakil F, Fribourg S, Minvielle-Sebastia L. 2011. An essential role for Clp1 in assembly of polyadenylation complex CF IA and Pol II transcription termination. *Nucleic Acids Res* **40**: 1226-1239.

Honorine R, Mosrin-Huaman C, Hervouet-Coste N, Libri D, Rahmouni AR. 2010. Nuclear mRNA quality control in yeast is mediated by Nrd1 co-transcriptional recruitment, as revealed by the targeting of Rho-induced aberrant transcripts. *Nucleic Acids Res* **39**: 2809-2820.

Janke C, Magiera MM, Rathfelder N, Taxis C, Reber S, Maekawa H, Moreno-Borchart A, Doenges G, Schwob E, Schiebel E et al. 2004. A versatile toolbox for PCR-based tagging of yeast genes: new fluorescent proteins, more markers and promoter substitution cassettes. *Yeast* **21**: 947-962.

Kim H, Erickson B, Luo W, Seward D, Graber JH, Pollock DD, Megee PC, Bentley DL. 2010. Gene-specific RNA polymerase II phosphorylation and the CTD code. *Nat Struct Mol Biol* **17**: 1279-1286.

Kuehner JN, Brow DA. 2008. Regulation of a eukaryotic gene by GTP-dependent start site selection and transcription attenuation. *Mol Cell* **31**: 201-211.

Longtine MS, McKenzie A, 3rd, Demarini DJ, Shah NG, Wach A, Brachat A, Philippsen P, Pringle JR. 1998. Additional modules for versatile and

economical PCR-based gene deletion and modification in Saccharomyces cerevisiae. *Yeast* **14**: 953-961.

Mischo HE, Gomez-Gonzalez B, Grzechnik P, Rondon AG, Wei W, Steinmetz L, Aguilera A, Proudfoot NJ. 2011. Yeast Sen1 helicase protects the genome from transcription-associated instability. *Mol Cell* **41**: 21-32.

Nagalakshmi U, Wang Z, Waern K, Shou C, Raha D, Gerstein M, Snyder M. 2008. The transcriptional landscape of the yeast genome defined by RNA sequencing. *Science* **320**: 1344-1349.

RCoreTeam. 2014. R: A Language and Environment for Statistical Computing. *R Foundation for Statistical Computing*.

Rondon AG, Mischo HE, Kawauchi J, Proudfoot NJ. 2009. Fail-safe transcriptional termination for protein-coding genes in S. cerevisiae. *Mol Cell* **36**: 88-98.

Sadowski M, Dichtl B, Hubner W, Keller W. 2003. Independent functions of yeast Pcf11p in pre-mRNA 3' end processing and in transcription termination. *EMBO J* **22**: 2167-2177.

Schmitt ME, Brown TA, Trumpower BL. 1990. A rapid and simple method for preparation of RNA from Saccharomyces cerevisiae. *Nucleic Acids Res* **18**: 3091-3092.

Schulz D, Schwalb B, Kiesel A, Baejen C, Torkler P, Gagneur J, Soeding J, Cramer P. 2013. Transcriptome surveillance by selective termination of noncoding RNA synthesis. *Cell* **155**: 1075-1087.

Stasevich TJ, Hayashi-Takanaka Y, Sato Y, Maehara K, Ohkawa Y, Sakata-Sogawa K, Tokunaga M, Nagase T, Nozaki N, McNally JG et al. 2014. Regulation of RNA polymerase II activation by histone acetylation in single living cells. *Nature* **516**: 272-275.

Steinmetz EJ, Brow DA. 1998. Control of pre-mRNA accumulation by the essential yeast protein Nrd1 requires high-affinity transcript binding and a domain implicated in RNA polymerase II association. *Proc Natl Acad Sci U S A* **95**: 6699-6704.

Steinmetz EJ, Warren CL, Kuehner JN, Panbehi B, Ansari AZ, Brow DA. 2006. Genome-wide distribution of yeast RNA polymerase II and its control by Sen1 helicase. *Mol Cell* **24**: 735-746.

Tan-Wong SM, Zaugg JB, Camblong J, Xu Z, Zhang DW, Mischo HE, Ansari AZ, Luscombe NM, Steinmetz LM, Proudfoot NJ. 2012. Gene loops enhance transcriptional directionality. *Science* **338**: 671-675.

Thiebaut M, Colin J, Neil H, Jacquier A, Seraphin B, Lacroute F, Libri D. 2008. Futile cycle of transcription initiation and termination modulates the response to nucleotide shortage in S. cerevisiae. *Mol Cell* **31**: 671-682.

Ursic D, Himmel KL, Gurley KA, Webb F, Culbertson MR. 1997. The yeast SEN1 gene is required for the processing of diverse RNA classes. *Nucleic Acids Res* **25**: 4778-4785.

van Dijk EL, Chen CL, d'Aubenton-Carafa Y, Gourvennec S, Kwapisz M, Roche V, Bertrand C, Silvain M, Legoix-Ne P, Loeillet S et al. 2011. XUTs are a class of Xrn1-sensitive antisense regulatory non-coding RNA in yeast. *Nature* **475**: 114-117.

Wickham H. 2009. *ggplot2: elegant graphics for data analysis.* . Springer New York.

Wilson SM, Datar KV, Paddy MR, Swedlow JR, Swanson MS. 1994. Characterization of nuclear polyadenylated RNA-binding proteins in Saccharomyces cerevisiae. *The Journal of cell biology* **127**: 1173-1184.

Xu Z, Wei W, Gagneur J, Perocchi F, Clauder-Munster S, Camblong J, Guffanti E, Stutz F, Huber W, Steinmetz LM. 2009. Bidirectional promoters generate pervasive transcription in yeast. *Nature* **457**: 1033-1037.

Yassour M, Kaplan T, Fraser HB, Levin JZ, Pfiffner J, Adiconis X, Schroth G, Luo S, Khrebtukova I, Gnirke A et al. 2009. Ab initio construction of a eukaryotic transcriptome by massively parallel mRNA sequencing. *Proc Natl Acad Sci U S A* **106**: 3264-3269.

Zhang Z, Dietrich FS. 2005. Mapping of transcription start sites in Saccharomyces cerevisiae using 5' SAGE. *Nucleic Acids Res* **33**: 2838-2851.
